# On the nature of grief: uncovering the evolutionary roots of loss across the animal realm

**DOI:** 10.1098/rstb.2025.0147

**Published:** 2026-09-24

**Authors:** Sarv Dashti, André Gonçalves

**Affiliations:** ^1^ Section of Cognitive Neuroscience, Kyoto University Center for the Evolutionary Origins of Human Behavior, Inuyama, Aichi Prefecture, Japan; 2 Science & Research Department, Indianapolis Zoological Society, Indianapolis, IN, USA

**Keywords:** comparative thanatology, non-human animal grief, dead conspecific, attachment and loss, animal mourning behaviour

## Abstract

Understanding grief in non-human animals poses conceptual and empirical challenges. Unlike humans, whose grief is readily identified through affective, cognitive and behavioural manifestations, recognizing it in other species is complicated by differences in expression and by anthropomorphic bias. Research on grief has taken several approaches. Observational studies in the ethological tradition have documented grief-like responses (withdrawal, vocalizations and changes in social interaction) across diverse taxa. Separation studies have shown that disrupting attachment produces depressive-like behaviour both behaviourally and physiologically, underlining how early bonds shape later affect. Questionnaire studies have explored perceptions of grief in companion animals, revealing both cross-species continuities and potential interpretive biases. Meanwhile, evolutionary hypotheses for grief range from non-functional accounts, through functional accounts (either cognitive or socially bound), to integrative/mechanistic models. Across these lines of evidence, we suggest that grief is the response to the rupture of a social bond built by a deeply conserved attachment system and its homologous nonapeptide toolkit. We propose an inclusive definition of grief as a response to the disruption of significant social bonds, encompassing affective, cognitive and behavioural components across species where the body is either present or absent. This definition is intended to ground systematic, comparative study of grief across species.

This article is part of the theme issue ‘Mechanisms, development, phylogeny and functions of emotional expressions’.

## 1. The nature of grief: theoretical and comparative foundations

**Figure 1.**
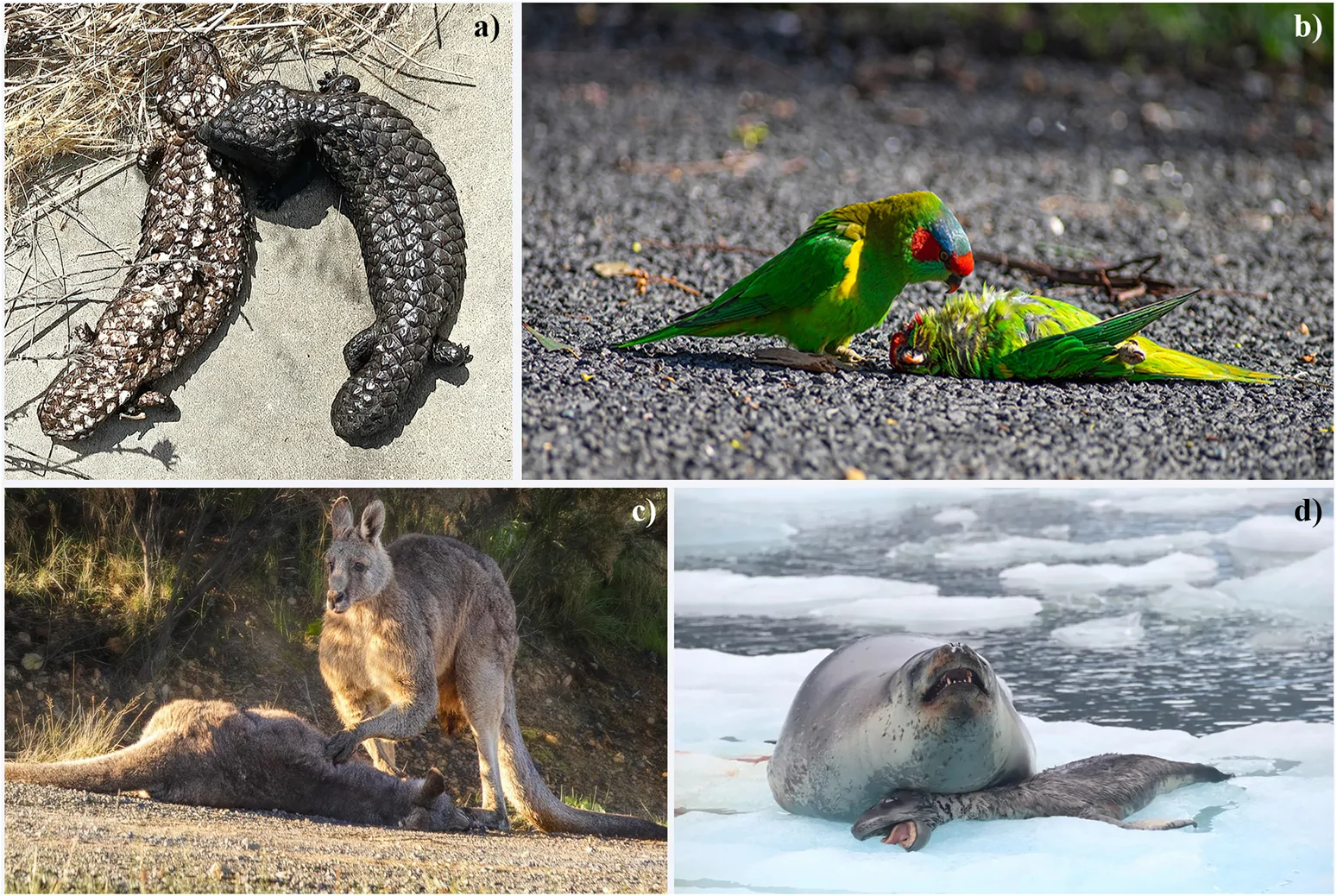
Physical interactions with deceased conspecifics across diverse taxa: (a) *Tiliqua rugosa* (sleepy lizard; Bristlenose), (b) *Trichoglossus concinnus* (musk lorikeet; Peter Murrowwood), (c) *Macropus giganteus* (grey kangaroo; Vicki Loyd-Smith and Pam Roxon), and (d) *Hydrurga leptonyx* (leopard seal; Renato Borras-Chavez). These interactions, often observed following the death of a bonded individual, are frequently portrayed in the media as evidence of grief. While these may depict the acute/protest phase of grief, the passive/despair phase is often under-reported.

Do animals grieve? Can they mourn their dead? These questions have been posited by naturalists from Aristotle to Darwin. Interactions towards the dead, such as physical contact or staying near deceased group members, are widespread across the animal kingdom and are of interest to comparative thanatology [1,2] (figure 1). Some such cases invite a ready attribution of grief. A recent event was when Tahlequah, a female orca whale, carried her dead calf for 17 days across more than 1000 kilometres [3]. This story drew worldwide empathy; her close bond with the calf and costliness of her behaviour made bereavement a plausible interpretation. King [4] argues that it is precisely this capacity to form close individual attachments, rather than brain size or cognitive sophistication, that determines which species can grieve. By this logic, similar corpse-directed interactions between individuals without close bonds are weak evidence of grief and may reflect general curiosity, confusion or being driven by perceptual cues. Experiments illustrated how baboons will show interest in and groom any fur-bearing object [5], and chimpanzees attend to conspecific skulls probably in response to face-like features rather than to death itself [6].

The more compelling signature of grief is what follows after a loss: the sustained changes in behaviour, often resembling anxiety/distress and depression, accompanied by comparable endocrine and neurobiological responses across species. The breaking of an existing bond, whether the loss of a parent, mate or social partner, can significantly affect an individual's fitness, and in response the bereaved individual may vocalize or search for the lost attachment figure. The loss of a parent or carer may be fatal for a dependent offspring. The loss of a bonded mate reduces parental investment and imposes the cost of finding a new partner. Moreover, the loss of an infant represents a direct fitness cost, and loss of a social companion can disrupt the cooperative social networks on which survival depends. Because pair-bonding is a central component of grief, the fact that it occurs in taxa outside birds and mammals, including some fishes [7] and reptiles [8], raises the possibility that analogous responses to partner loss may appear in these groups. However, whether such responses should be interpreted as grief rather than mate-guarding or other pair-bond-related behaviours remains unclear and needs to be further explored.

In this manuscript, we retain the term ‘grief-like’ throughout when describing the behaviours and grief when describing the phenomenon. Grief in the human sense is coloured by the cognitive representation of death as permanent and irreversible. Conversely, humans readily grant very young children the capacity to grieve despite them sometimes not having attained either a partial or full concept of death. However, for some animals, we use ‘grief-like’ not because we doubt that the underlying physical processes are testable (they are) or comparable (they appear to be), but because this cognitive dimension has not been established in other species.

In this review, we aimed to synthesize distinct schools of thought that all have contributed to expand our views on grief. From cultural anthropology, ethology/comparative thanatology, comparative psychology/affective neuroscience to social and evolutionary psychology, which in the past have not all been integrated into a single narrative. We then close by sketching a plausible evolutionary scenario for how grief has emerged in several taxa, including humans.

### (a) Darwin and the primate expression of grief

Charles Darwin was among the first to place grief within a comparative, evolutionary framework. He identified two states associated with grief: *excessive grief*, which he described as ‘frantic’ and ‘energetic’, and *sorrow*, which was ‘languid’ and ‘dull’ [9]. This dual active-passive character would later be rediscovered in the 1960s research on mother-infant separation in non-human primates [10] and formalized in evolutionary accounts of grief [11].

Darwin argued that tears and the characteristic facial contortion of grief were behaviours that originally evolved to protect the eyes during the more forceful infant crying. They were adaptive in their original context (e.g. signalling distress to carers) but persisted later into adult emotional displays.

Regarding the facial expression of grief specifically, Darwin described infants crying with closed eyes, wrinkled forehead and a widely opened mouth with retracted lips that gave it a square shape. He emphasized the downward slant of the eyebrows, produced by what he called ‘grief muscles’, and the downward turn of the mouth corners during intense distress [9]. Darwin speculated that this expression was unique to humans. He reasoned that the oblique eyebrow movement emerged only when screams are voluntarily suppressed. The muscles that contract to protect the eyes during crying are partially counteracted by antagonistic muscles that raise the brow, and it is this conflict that produces the characteristic appearance of sorrow. Since, according to Darwin, our early human ancestors had not yet acquired the habit of restraining their screams, this configuration would not have occurred [9].

Chevalier-Skolnikoff [12] reinforced Darwin's assertion that only humans shed tears, but identified similarities between human and ape sadness and crying expressions. In both, the lips are extended outwards and drawn down, the eyelids seem to droop and wrinkling appears near the outer corners and beneath the eyes. In the full cry expression, both species show an open mouth with a raised upper lip and a depressed lower lip. She also noted that if the muscular contractions which produce oblique eyebrows in humans were present in other great apes, these would produce a different appearance on their very differently shaped heads with little forehead. She observed some suggestion of eyebrow elevation and horizontal forehead wrinkling in apes appearing sad, and argued that the combination of formal and functional similarities (expressions occurring in similar contexts and ranging from mild sadness to intense crying in both species) was sufficient to suggest possible homology. She acknowledged, however, that whether ape whimpering and crying are truly homologous to human sadness and crying expressions remains unexplored [12].

Not all researchers are in full agreement; Preuschoft [13] noted that while chimpanzees produce vocalizations strongly reminiscent of crying or whimpering, these are combined with a lip configuration involving partially bared teeth, sideward-retracted mouth corners and protruded, puckered lips. According to her, that is not a configuration usually seen in humans. She stated that no precursors to sad expressions are documented in the monkey literature. In monkeys, the impression of a sick, lonely or hapless individual looking intensely sad is evoked primarily by slow movements and noticeably weak muscle tonus, instead of facial expression. Preutschoft concluded that it is questionable what communicative use a display of sadness would serve in the absence of a compassionate and caring social environment, suggesting that the evolution of sadness displays should be closely tied to the evolution of altruism.

Experimental work on how monkeys perceive facial expressions reinforces this picture. Kanazawa [14] found that a Japanese macaque (*Macaca fuscata*) categorized photographs of human ‘happy’ faces in a manner consistent with the morphological similarity between the human smile and the macaque silent bared-teeth display. Unlike humans, however, the monkey failed to differentiate ‘sad’ faces from ‘anger/disgust’ faces, lumping them into a single category. In a follow-up study, Kanazawa [15] used an odd-item visual search task with composite faces to identify which facial parts drove recognition in each species. For smiling faces, both species relied most heavily on the cheek region (the area activated by the *zygomatic major* muscle). For sad faces, however, results differed. Humans were most confused when distractors had either sad eyebrows or sad cheeks, indicating that both regions serve as important cues. Monkeys showed no effect for eyebrows. Kanazawa attributed this to the fact that macaques lack distinctly visible eyebrows (although they possess the same muscles) and, consequently, they lack the perceptual mechanisms tuned to detecting eyebrow orientation as a communicative signal. Whether this insensitivity is hard-wired or simply the absence of more relevant stimuli remains unexplored, as no follow-up study using monkey faces was conducted.

Anatomical studies by Waller and colleagues using intramuscular techniques have extended the basis of these species differences to chimpanzees [16]. Out of 20 facial muscles recognized in humans, 12 were successfully stimulated in anesthetized chimpanzees, with many of the same movements. The facial action coding system (a standardized facial coding system) is useful for this discussion as it breaks down facial expressions into discrete muscle movements called *action units* (AUs) [17]. The *zygomatic major* produced the same lip-corner-pulling action (AU12) in both species, confirming it as the likely muscular basis of the chimpanzee silent bared-teeth display. By contrast, the *corrugator supercilli* could not be shown despite detailed exploration. In humans, this muscle helps lower and knit the brows (AU4, associated with expressions of sadness, fear and anger). Despite this, subsequent dissections have confirmed it is anatomically present [18], but probably functionally reduced. Waller and colleagues argue that the human upper face appears more specialized for eyebrow movement, possibly because the retained hair covering on otherwise hairless brows has increased their signal value [16]. Furthermore, the *triangularis* muscle, historically termed the muscle of sadness associated with a downturned mouth, was successfully activated in chimpanzees, causing the lip corners to lower (AU15). Later, Vick *et al.* [19] would claim that despite anatomical similarities, no clear spontaneous examples of AU15 have been documented in chimpanzees. Similarly, Kavanagh *et al.* [20], reviewing primate facial expressions in primates, also note that the AU15 activated by the *triangularis* appears unique to humans.

What these studies suggest is that although the facial musculature underlying emotional expression is highly conserved across great apes, the specific configurations associated with human sadness and grief may not have direct equivalents in other species. The eyebrow region that is so central to the human expression of grief appears to lack the muscular independence, the signal value and the corresponding perceptual recognition mechanisms in non-human primates. It remains unclear whether apes have structurally distinct but functionally related sadness facial expressions [12], or whether observers interpret as behavioural or bodily cues as sadness [13]. However, for researchers, the behavioural and physiological responses to loss (e.g. withdrawal, reduced activity, disturbed sleep and appetite in addition to elevated stress hormones) have been more reliable cross-species indicators of grief.

## 2. Empirical studies on animal grief

### (a) Primate separation studies, an outline

In the 1960s, studies on mother-infant separation in non-human primates [10] described two states: *protest* and *despair*. While *protest* was characterized by marked increases in movement and vocalizations, *despair* was characterized by marked decreases in activity and increases in self-directed behaviours such as huddling and self-clasping. Importantly, *despair* is not fatigue; separated infants respond rapidly when food is presented or aggression directed at them, and return to normal activity upon reunion. Both states are accompanied by changes in body temperature, heart rate, endocrine and immune function, neurochemistry and sleep patterns, together with decreases in food and water intake; in some cases, separation has been followed by death [10].

This research had its main inspiration in human medical reports. During the 1940s, work by Harry Bakwin, William Goldfarb and René Spitz showed how early separation and hospitalization negatively impacted infants. Their findings influenced Harry Harlow, who began the mother-infant separation studies in rhesus macaques, and John Bowlby, who formulated the attachment theory on which subsequent studies are grounded [21]. Bowlby described *protest*, *despair* and *detachment* in separated human children [22,23]. However, while *protest* and *despair* appear consistently among primates, *detachment* is uncommon, and reunions usually prompt immediate attachment behaviours [24].

Not all infants show the full response either, and those that do, vary widely in severity. In Spitz's original study, only about 25% of separated infants exhibited the full syndrome: a convergent estimate also obtained for non-human primates [25,26]. Surprisingly, age and sex seem unimportant within the first year, with the variables that matter being relational (attachment bond) and environmental (familiar versus strange place) [24]. Anxiously attached infants (e.g. those spending the most time maintaining maternal proximity) show the most severe protest-despair reactions [27]. The availability of alternative attachment figures is critical: pigtail macaque infants (*Macaca nemestrina*), whose possessive mothers restrict social contact, reliably show severe depression upon maternal removal, whereas bonnet macaque infants (*Macaca radiata*), reared in permissive groups with multiple ‘aunts’, redirect attachment behaviours and rarely exhibit *despair* [28,29]. Crucially, when the pigtail infant was adopted by a familiar female with whom it had no prior attachment, the response was not ameliorated [25,30]. When social networks were reduced experimentally in bonnet infants through environmental manipulation, they showed depressive reactions usually absent in this species [25]. Similarly, unlike infants that retained their affiliative partners, unsupported infants exhibited significant immune suppression (reduced lymphocyte proliferation and natural killer cell cytotoxicity).

The physiological consequences observed in non-human primates are extensive. The *protest* or *agitation phase* is marked by hypothalamo-pituitary-adrenal (HPA) activation, which shows itself as an increase in cortisol, and is shaped by peer availability, separation length and environmental novelty [31]. In squirrel monkeys (*Saimiri scuireus*), cortisol continued to rise even as vocalization declined, reflecting different timescales among behavioural and endocrine responses [32]. Biotelemetry measures in pigtail macaques showed an initial increase followed by a decline in heart rate. The animals also exhibited heart rhythm disturbances and disrupted sleep during the despair phase [29,33]. Mother-offspring separation lowered lymphocyte responsiveness, which returned to baseline after reunion. Moreover, *in vivo* assessment showed diminished antibody responses in separated infants immunized with foreign proteins [32,34,35]. Peer separation produced similar immunological changes, ruling out nutritional deprivation as an explanation. In free-ranging rhesus macaques (*Macaca mulatta*), behavioural variables during naturalistic separations (i.e. first-day vocalizations, slouched postures and social contact) predicted more than 50% of the variance in immune measures [36]. Baseline heart rate predicted both behavioural and immunological vulnerability, suggesting autonomic reactivity as a biological marker for susceptibility to the consequences of bond disruption [25].

Separation left lasting marks: adult pigtails separated at six months old showed, at four years, smaller social networks, greater behavioural disturbance and reduced immune responses [32,37]. Hinde & Spencer-Booth [27] found effects that were detectable two years after reunion. Early separation also sensitized animals to later loss. Gilmer & McKinney [10] termed these ‘scars’, describing them as increased vulnerability that emerged only under the stress of subsequent separation. Rearing history only mattered under stress: peer-reared and mother-reared monkeys looked similar at baseline, but peer-reared monkeys showed more disturbance during separations [38]. Pharmacological findings support this, as antidepressants such as imipramine, fluoxetine and desipramine all reversed or prevented the separation response, with latency periods matching the human clinical timeline [10].

Thierry and colleagues' [39] *searching-waiting strategy* offers a unifying framework across different perspectives. It reframes the *protest-despair* dyad as a continuum across which behaviour is shifted according to continually changing circumstances, leading to updated cost-benefit assessments. On this view, clinical depression does not reflect this strategy but a pathological inability to switch between waiting and searching when circumstances alter. When reunion seems likely, the infant searches actively (higher locomotion and vocalizations to draw attention); when it seems unlikely relative to the energy cost, the infant shifts to waiting (conserving energy and reducing predation detection risk) [28,29]. This accounts for otherwise puzzling findings: infants removed to novel environments show prolonged *protest* but delayed *despair* (searching for home range/familiar environments is the effective strategy), while infants remaining in the home cage with the mother removed show rapid onset of *despair* (waiting for the mother's return is more effective) [40].

McKinney [41] defined four criteria an animal depression model should satisfy: etiological correspondence (the same inducing conditions cause the response), symptomatic correspondence (the same behavioural states observed), mechanistic correspondence (the same underlying neurobiology detected) and therapeutic correspondence (reversal by the same clinically effective treatments). Many of the species covered here pass McKinney's first two criteria: a shared precipitant (loss of an attachment figure) and a shared behavioural response (protest then despair) are recognizable across many social species. The therapeutic criterion may hold among social mammals in which the response draws on the same conserved attachment machinery [10]. In addition, the finer neurobiology depends on more species-specific detail, and so corresponds most readily among closer relatives while diverging in more distant ones.

Bonanno & Kaltman [42] describe distinct types of disrupted functioning in bereaved humans that map closely onto the primate data, such as *cognitive disorganization*, *dysphoria*, *social withdrawal*, *loss of appetite*, *insomnia* and *altered neuroendocrine and immune functioning*. It is telling that a brief maternal separation in an infant monkey (in the absence of confounding health factors that inevitably complicate human bereavement research) produces the same constellation of changes seen in bereaved human adults. Such a parallel provides compelling evidence for the phylogenetic conservation of the response to bond loss [32].

### (b) Neurobiological effects of separation in pair-bonded species

#### (i) Partner-seeking behaviour following loss

When bonded male prairie voles are separated from their female partners, they will shift to actively searching for them. Vitale *et al.* [43] used an odour-preference test and found that males separated from female partners investigated partner-associated odour more than three control groups: males separated from male cage-mates, males kept with their partners and unpaired males. However, this increase was specific to the partner's odour; when stranger-scented bedding was substituted, no differences were observed. Sadino & Donaldson [44] note that this is yearning (*sensu* [45]), an emotionally painful desire to re-unite with the lost individual, reflecting continued activation of reward circuits that previously maintained the bond. Yearning (being tied to the *protest phase*) is a core feature that distinguishes grief from other negative emotional states such as depression, and (perhaps being distinct from the *despair phase*) it is not ameliorated by standard antidepressant pharmacotherapy.

However, this partner-seeking behaviour depends on the quality of the bond. Approximately 30% of male prairie voles housed with females do not form a selective partner preference. Vitale *et al.* [43] found that only pair-bonded males showed increased partner odour investigation after separation; non-bonded males did not. Vitale *et al.* [46] extended this finding, showing that only pair-bonded males maintained a socially conditioned place preference for the partner-associated context after separation, and only pair-bonded males showed increased passive stress-coping in the *forced swim test*. The bond-quality dependence also extends to somatic domains: anxiety was higher in bonded males who lost their partners, and so was sensitivity to noxious stimuli and lower thermal thresholds, which correlated with anxiety [47].

Sex differences in partner-seeking were also reported. Female voles whose male partner were removed showed increased investigation of partner- and food-associated cues, itself driven by dopamine receptor type 1 (DRD1) signalling in the *hypothalamic medial preoptic area* [48]. Previous work in the same laboratory had found DRD1&2 changes in the *anterior insular cortex* (aIC) and *anterior cingulate cortex* (ACC) for males [43]. Thus, although the neurochemical substrates are sexually dimorphic, the search behaviour is conserved across sexes.

#### (ii) Neural and physiological mechanisms of the grief-like state

The mechanistic pathway underlying the grief-like state has been traced most completely by Bosch and his colleagues. Bosch *et al.* [49] were the first to demonstrate that separation from a female partner, but not a male sibling, resulted in increased passive stress coping together with elevated basal corticosterone levels and increased adrenal hypertrophy. Pair bonding did, however, increase *corticotropin-releasing factor* (CRF) messenger RNA (mRNA) levels in the *medial bed nucleus* of the *stria terminalis*, suggesting that bond formation sensitizes the CRF system to subsequently respond to separation. Blocking type 1 and type 2 CRF receptors during separation eliminated the passive coping response but did not disrupt the partner preference. In coyotes, monogamous pair-bonded mammals with a considerably longer lifespan than prairie voles, similar CRF-related changes were observed. Specifically, partner loss produced region-specific differences in CRFR1 and 2 bindings, particularly in the olfactory bulb, suggesting this pathway is not only confined to rodents [50].

Bosch *et al.* [51] located this pathway within the *nucleus accumbens* (NAc) shell. In that region, dopamine signalling is required for bond formation, and oxytocin (OT) receptor activation facilitates partner preference. The loss of a partner activates the CRF receptor type 2 (CRFR2) on OT fibres projecting from the *paraventricular nucleus* (PVN) to the NAc shell, in turn suppressing local OT release. At the same time, OT mRNA in the PVN decreases, and OT receptor (OTR) binding in the NAc shell is reduced. Such suppression at multiple levels of OT signalling produces the passive coping behaviour. Chronic OT infusion into the NAc shell reversed this passive response to normal behaviour. Strikingly, even in still-paired males, the OTR knock-down via short hairpin RNA in the NAc shell mimicked partner loss.

Bales & Rogers [52], drawing on *positron emission tomography* imaging of separated male titi monkeys, proposed that the *kappa opioid receptor* (KOR) system acts as an intermediary in this pathway. Upon acute separation, CRFR2 activation promotes dynorphin release and activates KORs, which in turn inhibits OT release. During long-term separation, KOR inhibition in the PVN may decrease; in that scenario, OT escapes suppression and can drive social seeking. This model integrates the CRF and OT findings with the well-established role of the KOR system in dysphoria and aversion. Separated males showed less glucose in the *ventral pallidum*, *lateral septum*, the PVN and the *periaqueductal grey,* plus elevated cerebrospinal fluid OT levels during both short- and long-term separation, while plasma OT rose only on reunion with partner.

The dopamine system is also remodelled by partner loss. Vitale *et al.* [43] found that pair-bonded vole males who lost their partner showed elevated DRD1 and DRD2 mRNA in the aIC and ACC, the same regions that show heightened activation in functional magnetic resonance imaging studies of grieving humans viewing photographs of the deceased [53,54]. Non-bonded vole males showed no such changes.

In zebra finches, another lifelong monogamous species, Remage-Healey *et al.* [55] showed that separation from the bonded mate elevated plasma corticosterone. The same was true in conditions where male birds were housed with same sex individuals; therefore, the effect of separation is not a result of general social isolation. It was only reunion with the former mate that reduced corticosterone to baseline levels within 48 hours, while introducing a novel opposite-sex individual did not. Thus, the recovery was specific, not to the sex alone, but to the identity of the bonded partner.

In free-living greylag geese, Ludwig *et al.* [56] tested the same responses to social loss. The researchers removed males from eight pair-bonded couples for a 48 hour-period, leaving females in the flock while males were placed in isolation aviaries. Isolated males showed the full physiological response with elevated corticosterone metabolites, decreased haematocrit and elevated leucocyte numbers and heterophil/lymphocyte ratio. Females, who remained embedded in their normal social environment, showed behavioural searching, with a tendency towards increased vigilance and distance calling, a significant haematocrit decrease, but no significant overall rise in corticosterone metabolites. In both sexes, an elevated coccidian oocyst excretion was observed at 48 hours and one week, then returning to baseline by four weeks. The dissociation between persistent behavioural searching and peripheral stress markers on the one hand, and dampened HPA activation on the other, in the flock-embedded females extends earlier findings from the same flock that family members buffer corticosterone responses to social stressors in greylag geese [57].

The HPA response drives the neuroendocrine signature, and is specific to mate loss rather than social loss in general, mirroring findings in titi monkeys where mate separation elicits cortisol elevation but separation from other family members does not [58]. Returning to prairie voles, partner loss also affects the brain's immune and structural architecture beyond neuromodulatory systems. Pohl *et al.* [59] observed that partner loss resulted in increased microglial priming in the parvocellular PVN in males while leading to decreased microglial activation in the *prelimbic cortex* and the PVN in females. Sadino *et al.* [60] discovered a stable transcriptional signature in the NAc associated with pair bonding, composed of gliogenesis, myelination and extracellular matrix organization. These structural changes, which were unprecedented in the current literature on grief, do erode after prolonged separation, potentially reflecting recovery on a molecular scale.

#### (iii) Loss adaptation and recovery

Sun *et al.* [61] reported that male voles maintained a partner preference for up to two weeks of separation, but after four weeks at which time they became affiliative towards strangers too and ceased intruder-directed aggression. Sadino *et al.* [60] show that the NAc transcriptional signature is largely unaffected by 48 hours of separation but almost fully eroded at four weeks, by which time males again formed a new bond [62]. In the wild, after losing a mate, male voles remain an average of 17 days at a burrow before locating another mate [63]. These results converge across laboratory and field work and were incorporated into a reward-modelling framework by Sadino & Donaldson [44]. Pair bonding causes long-term changes in the NAc reward circuitry: greater partner-associated dopamine release and partner-associated neuronal ensembles, along with persistent shifts in gene expression. Loss adaptation involves the gradual erosion of these changes; for instance, partner-associated dopamine enhancement weakens, the transcriptional signature reverts and OT immunoreactivity in the PVN returns to pre-bonding levels. Consistent with partial erosion of the bond without forgetting the partner, behaviours such as reduced partner-directed huddling but increased investigation of both familiar and novel individuals are expressed [44].

The authors propose that *prolonged grief disorder* (PGD) may result from stalling of this remodelling process. In humans, PGD is associated with sustained NAc activation in response to reminders of the deceased, whereas normal grief resolution corresponds with decreased NAc activity [54]. A striking feature of the Sadino *et al.* [60] data is that transcriptional erosion substantially precedes behavioural dissolution: the NAc molecular signature had all but vanished by four weeks, yet partner preference persisted, suggesting signatures may occur elsewhere in the brain. These results mirror the research on human grief resolution, in which the capacity to form new attachments emerges while there is still an emotional connection to the deceased.

Pair-bonded birds also show patterns of bond persistence followed by eventual dissolution. Budgerigars resume the pair bond after a period of up to 70 days of separation in unisexual groups, still recognizing their pair-mates on an individual level rather than choosing them by general quality [64]. When paired for an equal number of days with two different consecutive mates, budgerigars preferred the more recent mate, which would suggest that formation of a new pair bond erodes an older one more effectively than time on its own, consistent with the reward modelling framework. Separated zebra finches retain previous bonds by auditory contact alone. However, after total isolation, new pair bonds are formed almost immediately. Notably, formation of a new bond does not necessitate the original one being destroyed: all 12 experimental pairs that were paired again after 14 days of total isolation from their mate resumed the original bond upon reunion [65]. Lastly, Eda-Fujiwara and colleagues [66] demonstrated that female budgerigars continued to respond preferentially to their mate's contact call two months after separation, with the preference disappearing by six months.

In all, the fact that the same behavioural and physiological patterns appear in both mammals and birds initially suggests that the neuroendocrine response to attachment disruption is either deeply conserved or convergently evolved across pair-bonding vertebrates. The current picture points to a more complicated picture: a convergence on the pair bond and caretaking behaviours with a deeply conserved molecular toolkit of nonapeptide and dopaminergic systems [67].

### (c) Empirical evidence following natural deaths in primates

Cheney & Seyfarth [68] observed that monkeys never consoled others after their loss. They attributed this absence to a possible limitation in monkeys' capacity to attribute mental states to others. Later, Seyfarth & Cheney [69, p. 342] extended the observation to chimpanzees: ‘*although chimpanzees have mental states and grieve at the loss of close friends, they do not seem to recognise the same mental states in others. As a result, they are unable to share another's sorrow or show empathy towards it*’.

A long-term study on free-ranging chacma baboons (*Papio h. ursinus*) in which predation by leopards and lions was the primary cause of adult mortality, over a 16-month period, found 23 individuals were confirmed or suspected to have been killed. Engh *et al.* [70] collected faecal samples to measure glucocorticoid (GC) levels (a stress indicator), and recorded grooming behaviour of the female baboons. A total of 22 females who lost a close relative (mother, maternal sibling or offspring) experienced a significant increase in GC levels during the month after the death compared with the month before. Matched control females whose relatives did not die showed no such increase. Thus, the effect was not simply a response to witnessing a predation event: predator attacks were observed by many adult females, but only those who lost a close relative showed elevated GC levels. Although female baboons concentrate much of their grooming on close kin, bereaved females did not show a decrease in grooming following a relative's death. The opposite occurred: grooming diversity, number of partners (from an average of 1.86 to 3.79) and grooming rate, all increased significantly in the three months following the death. Control females showed no such changes. The rise in GC levels lasted only for a while: by the second month after loss, levels had returned to baseline. Engh *et al.* [70] concluded that females compensated for the loss of a specific companion by broadening and strengthening their social relationships/grooming networks. Crucially, however, the social initiative was unidirectional: it was the bereaved females seeking new grooming partners rather than other group members spontaneously offering consolation.

Seyfarth & Cheney [71] analysed the behaviour of 45 females over a 7-year period. Using principal component analysis, they identified three stable personality dimensions: nice females, who were friendly to others and often grunted to lower ranks to signal benign intent; aloof females, more aggressive, less friendly and grunted mainly to higher-ranking females; and loner females, who were relatively unfriendly and often seen alone. Nice females had the highest social bond strength and the most stable partner preferences. By contrast, loner females had the weakest bonds, least stable preferences and significantly higher baseline GC levels. Reanalysing the bereavement data by personality type highlighted stark differences in the behavioural response to loss. Nice and aloof females had more grooming partners than unaffected females in the three months following a close relative's death, consistent with actively rebuilding their social networks. Loner females, failing to compensate for their loss, had fewer grooming partners than unaffected females, suggesting they did not rebuild their social ties. Moreover, they also tended to show greater GC increases after losing a mother or adult daughter, despite being the least successful at establishing replacement bonds. Moreover, such findings parallel the human clinical research on personality and grief resilience.

In southern India, Arlet and colleagues [72] observed behavioural changes across 18 wild bonnet macaques before and after the deaths of their infants. While only two persistently carried their dead infants, most bereaved mothers became more peripheral, had fewer near neighbours and received less grooming. Additionally, they initiated less hugging, showed more stress-related behaviours (e.g. scratching and yawning), vocalized more (contact calls) and directed more aggression towards other group members than before. The authors interpreted these changes as consistent with grief. Carter & Huchard [73] predicted that if grief drives social withdrawal, then the mother should initiate less grooming but continue to receive the same amount; if natal attraction loss is responsible, the mother should initiate the same amount but receive less. Arlet *et al.*'s [72] data fitted the second prediction. Carter & Huchard [73] recommended comparing bereaved mothers with matched controls who also lack infants, as Engh *et al.* [70] had done, to isolate the internal emotional response from the external social consequences of infant loss.

At Cayo Santiago, Johnson and colleagues [74] followed 11 bereaved free-ranging rhesus macaque mothers together with 11 matched controls (unrelated females of similar age without infants from the same group). They tested four predictions derived from the human grief literature: bereaved mothers should spend more time resting (lethargy), less time feeding, more time in displacement behaviours (stress) and less time grooming. Against their expectation, bereaved mothers tended to rest less than controls in the first two weeks. However, behaviours like feeding, grooming or displacement were not significantly different at any point in time. The authors believed this increased activity reflected the *protest phase* of grief rather than the *despair phase* they had predicted. They noted that the few studies on primate mothers in captive scenarios generally do not seem to reliably enter the despair stage after their infants have been removed, in contrast to the primate infant literature (§2a). For future studies, they proposed a few revised predictions. Namely, they distinguished an initial period of protest grief (marked by increased activity, behavioural state changes and vocalization) from a subsequent period of despair grief (marked by lethargy, reduced appetite and social withdrawal) that may or may not follow, depending on the individual. The infants were also exceptionally young (median age: 9 days), which may have limited the strength of the mother-infant bond and consequently, the intensity of their response. Johnson *et al.* [74] also acknowledged the possibility that macaque mothers may not grieve in a manner homologous to humans.

At Burgers' Zoo, Goldsborough *et al.* [75] recorded the behaviour of 15 adult chimpanzees after Moni, a recently introduced female, had a stillborn infant. The study drew on six months of structured observations before and after the event for quantitative comparison. The affiliative interactions Moni received showed a pronounced and selective increase following her loss (a 210% increase from January to February), while most other group members showed no comparable change. Five individuals who had shown no affiliation with Moni in the month before the event began showing affiliative behaviour towards her, a pattern not found in other group members. Curiously, the highest increases came from Tushi (who had herself experienced a stillbirth in 2004), Morami and Ghineau. Moni received species-typical reassurance behaviours rarely observed outside this context (e.g. body kisses, mouth-to-mouth contacts and finger-in-mouth gestures), mainly on the day of the event and the day following. These behaviours are characteristic of reconciliation and consolation contexts in chimpanzees and are considered markers of empathy [76]. The authors also ruled out many alternative explanations. The increase was not explained by reproductive state: Moni received less affiliation when pregnant, and her first post-event swelling occurred two months later. It did not stem from a natural group integration process: the other newly introduced female, Erika, followed a different affiliation trajectory. It was not explained by enclosure location or curiosity towards the corpse, which was not present during any observations used in the model. Crucially, the other group members approached the bereaved mother, not the reverse. Moni did not initiate or solicit what became an increased social interaction. In contrast to baboons' self-directed coping, this represented other-directed consolation. The following social effects were also notable. Another individual who showed a marked increase in received affiliation, Jimmie, received most of that from the kin of the two females most invested in consoling Moni. Tushi and Morami shifted their affiliative attention towards the bereaved mother, so their usual partners compensated by affiliating more with Jimmie.

Taken together, these naturalistic studies reveal a gradient in the social response to bereavement across primate species. In monkeys, bereaved individuals show measurable stress responses and, in some cases, actively compensate by seeking new social partners. Sustained consolation of the bereaved by group members, however, has not been systematically documented. Challenging previous claims, chimpanzees do show increased affiliation and reassurance behaviours towards the bereaved, suggesting a capacity for empathetic response to others' loss that may be more readily expressed in species with greater social tolerance and emotional complexity [77]. Looking forward, third-party affiliation has been observed across several taxa [76], and some of these may be good candidates for grief-directed consolation behaviours.

## 3. Companion animal grief: public attitudes, owner reports and veterinary perspectives

### (a) Public attitudes towards animal grief

Morris *et al.* [78] conducted a study systematically investigating secondary emotions across non-primate species. Conducting their survey in the UK, they asked pet owners (*n* = 907) about the emotions they observed in their pets. All owners had a minimum of two years' experience with their pets (which included dogs, cats, horses, birds, rabbits, rodents, etc.). They found that primary emotions (e.g. fear, joy, sadness and anger) were reported far more frequently than secondary emotions (e.g. grief, jealousy, guilt and pride), and that owners were significantly more confident about primary emotion attributions. Grief was reported at notable levels across taxa: 49% of dogs, 47% of horses, 46% of birds, 43% of rats, 40% of cats and 37% of guinea pigs, with lower rates in rabbits (25%) and hamsters (23%). However, the differences among species were smaller for grief than for other emotions such as jealousy or guilt, which in turn suggests owners were more willing to attribute grief across a broader range of species. Importantly, these authors did not report owners' gender, which limits interpretation, given later work showing gender may affect emotional attribution. While anthropomorphic bias cannot be ruled out, these findings nonetheless suggest such emotions may be present in animals.

A survey by McGrath and colleagues [79] of Australian residents in Brisbane (*n* = 999) found that 90% thought some animals or all could grieve (67% believing some and 23% believing all could, while the remaining 10% believed none). Respondents attributed grief most readily to species perceived as cognitively complex and socially bonded: dogs (98%), chimpanzees (97%), dolphins (94%) and elephants (94%), followed by cats (88%), pigs (73%) and cows (71%), with much lower rates for chickens (40%), fishes (19%) and invertebrates such as ants (18%) and prawns (10%). Over 70% attributed grief to cows and pigs. While these species show separation distress, long-term studies documenting the *protest-despair pattern* are lacking [79,80]. The study showed both age- and gender-related differences: women were significantly more likely than men to attribute grief to most species, while older respondents were less likely to attribute it to several species [79].

Using the same sample as McGrath *et al.* [79], Walker *et al.* [81] found that 66% of respondents believed animals experience grief in the same way as humans. Pet owners tended to endorse animal grief, while non-owners were roughly three times more likely to deny animal emotions and about four times more likely to be unsure [81]. The high percentages among those who disagreed (91% stating animal grief is less intense and 97% stating it was shorter in duration) indicate most dissenters judged animal grief as weaker than human grief, implying they made a measured distinction rather than simple projection. Both groups recognized specific scenarios tended to produce grief at high rates, citing parent-offspring separation and loss of a mating partner each at 93%. A follow-up study found that men were significantly less likely than women to attribute grief, depression, anxiety and love to animals, while neither gender differed in their attribution of basic emotions such as fear, happiness or sadness [82]. They note people who have experienced grief are more likely to attribute it to animals.

### (b) Owner-reported behavioural changes in companion animals

Walker *et al.* [83] surveyed companion animal owners in Australia and New Zealand whose dog or cat had lost a companion. Of 414 surviving animals, 75% (*n* = 311: 159 dogs, 152 cats) showed at least one behavioural change, with a mean of nearly five changes per animal. Dogs were reported to eat less (35%), eat more slowly (31%), sleep more (34%) and seek more affection from their owners (35%). Cats were reported to vocalized more often (43%) and more loudly (32%). Both dogs (30%) and cats (36%) sought out the deceased favourite area. Changes in affectionate behaviour lasted from two to six months. Although 73% of surviving animals sniffed and investigated their companion's body when shown it, viewing the body had no significant effect on subsequent behavioural changes. The respondent sample was heavily skewed towards women (91%), consistent with the gender effects documented by Walker *et al.* [82]. Because of the self-selecting nature of the sample, owners who noticed changes were more likely to respond.

Uccheddu *et al.* [84] cross-referenced owner grief with reported behavioural changes in surviving dogs (*n* = 426; 384 women, 42 men) after the loss of a companion dog in an Italian online sample. The surviving dogs sought more attention (67%), played less (57%) and showed lower activity (46%). Moreover, they slept more (35%), increased in fearfulness (35%), ate less (32%) and vocalized more (30%), with roughly 13% of owners reporting no change. Among dogs, it was relationship quality, not length of cohabitation, that best predicted the severity of these changes. A friendly relationship predicted decreases in play, a parental relationship predicted decreased appetite, increased fearfulness and increased vocalization, and food-sharing predicted reduced activity and increased sleep. Importantly, owners' general attitudes towards animals, including humanization, attachment to pets and views on human-animal continuity, did not correlate with any reported canine behavioural changes. This suggests that owners were not simply projecting their own grief onto their dogs. Although their findings are compatible with the pattern of loss responses, they note they could not confirm if this constituted grief. They explain the behavioural changes were equally consistent with separation distress as viewing the body had no effect on the surviving dog's behaviour. This, they suggest, was a response to absence rather than death, and the influence of owner emotional state on some of these dogs could not be fully ruled out.

Greene & Vonk [85] conducted the first systematic study on domestic cats' responses to the death of a companion animal. They surveyed carers in the United States (*n* = 412; 340 women, 72 men) about their surviving cats' behaviours. Unlike previous studies, they had owners rate behavioural changes in a five-point scale, so results are reported as directions of change rather than prevalence percentages. Their findings indicated that, like dogs, cats showed alterations in their behaviour following the loss of a peer. These changes included reduced appetite, sleeping and playing. There were also increases in attention-seeking, vocalization, hiding and apparent search for the lost companion. These changes correlated with the strength of the bond and amount of time spent together in daily routines. Moreover, they note that cats reacted in much the same way whether they lost a dog or a cat companion. There is a possibility some of these reports may have been influenced by anthropomorphism, especially with regard to the emotional states and attachment styles. The authors state that carers experiencing deeper grief reported increases in sleeping, hiding and time alone, while those more attached reported more attention seeking. Conversely, those with avoidance attachment towards their pet reported fewer grief-like responses.

Ricci-Bonot *et al.* [86] surveyed horse owners, primarily in the UK, about behavioural changes observed in 325 surviving horses after the loss of a companion equid (*n* = 338; 329 women, nine men). Within 24 hours following the loss, owners reported changes in arousal (89%), behaviour directed towards people (78%) and other equids (78%), alertness to stimuli (73%), vocalization (69%) and decreased feeding (62%). Moreover, these changes were more pronounced in horses sharing a friendly or parental-dependent bond with the deceased. While the authors noted that many of these responses resembled those observed during temporary separations, they state that horses which witnessed the death showed additional changes in feeding, sleeping and vigilance. These marked reactions not seen during routine separations suggest a response beyond separation distress. Perhaps the most intriguing discovery pertained to access to the corpse. While viewing the body had no immediate impact on behaviour in the first 24 hours, horses who spent time with the corpse showed fewer long-term changes in vocalization and arousal throughout the ensuing six months. By contrast, horses prevented access displayed enduring changes in their arousal and vigilance. The researchers suggested that proximity to the body might assist horses in processing the permanence of loss, despite that witnessing death itself might produce greater immediate distress. Unlike Uccheddu *et al.* [84] and Greene & Vonk [85], this study did not measure owner grief or humanization, so the influence of owner emotional state on reported horse behaviour could not be assessed.

Overall, the same pattern recurs across species, countries and independent research groups: relationship quality predicts the response to loss, pointing to companion animals reacting to the loss of a social bond rather than a mere change in routine or owners' projection. Future work would benefit from controlled designs comparing temporary separation with permanent loss, pre-loss baselines and objective measures such as changes in eating, sleeping and activity [74,87,88].

## 4. Evolutionary accounts of grief and depression

Throughout this paper, we have documented grief-like responses across a range of taxa, and a convergent picture emerged: bond disruption produces a conserved behavioural response characterized by initial agitation, searching and vocalization, followed by withdrawal, reduced feeding and social disengagement. While there are many definitions of grief, these often describe separation responses observed in the laboratory and fail to capture what is documented in naturalistic settings where individuals can interact with dead conspecifics, sometimes for extended durations. We propose a definition that integrates both:

Grief is the prolonged behavioural, physiological and affective response of an animal to the disruption of a close social bond (kin, mate or peer), whether through separation or death. The response has three characteristic features. First, an active phase of distress, including vocalization, search behaviour and, where the body is present, sustained attempts to re-engage the deceased or maintain proximity to the body. Second, a passive phase of withdrawal, usually following, sometimes interwoven with the first, characterized by reduced feeding and activity, altered sleep and elevated stress, which persists in the absence of reunion or in the continued presence of an unresponsive partner. Third, resolution, occurring through the formation of new bonds or the gradual updating of the lost individual as no longer a viable social partner.

This definition is most fully instantiated in laboratory models and captive studies detailing mate and maternal bond disruption, where all three phases can be tracked longitudinally. Outside the laboratory, the picture is complicated by the ethological data. First, the *passive phase*, where it appears, is neither universal nor always internally generated. The *passive phase* following the *protest phase* is more diagnostically meaningful than *protest* alone, but is not always visible: social context can suppress it, *protest* can persist in its place, or environmental disruption can produce similar withdrawal without grief. Second, the *resolution phase* need not involve social substitution. In ‘solitary species’, where mate and mother-offspring bonds form without embeddedness in a wider group, resolution proceeds through gradually updating the internal representation of the lost individual rather than through re-affiliation with available conspecifics. Longitudinal monitoring of known individuals in such populations could, in principle, supply the passive-phase and resolution data. Still, in general terms, we find that applying McKinney's depression criteria to grief, there is substantial evidence for all four in controlled studies: *identical aetiology*, *similar symptomatology*, *comparable neurobiology* and *corresponding treatment attenuation* [41].

While never formulating a hypothesis himself, Darwin [9] argued that emotional expressions, including grief, are shared across species through common descent and differ in degree rather than kind. The comparative evidence reviewed above is broadly consistent with this premise. The question that has occupied subsequent research is not whether a homologue of grief exists in other animals, but why it exists at all.

Evolutionary accounts of sadness and depression are numerous and intersect with the literature on grief at several points. While grief recruits sadness and depression as component processes, it is not reducible to either, and our focus here is on hypotheses that address the grief response specifically. These grief-specific hypotheses differ in whether they view grief as non-functional (a by-product of adaptive attachment mechanisms), as functional in a cognitive sense (improving future decision-making), as functional in a social sense (influencing the behaviour of others), or as the output of phylogenetically ancient mechanisms whose adaptive significance depends on context. Table 1 summarizes the major hypotheses, their proposed functions, predicted species range and key references. For more information, see the supplementary material.

**Table 1.** Evolutionary accounts of grief across functional and phylogenetic categories.

category	hypothesized function	description	predicted species range	key references
**non-functional (by-product)**	separation response	grief is the activation of a separation response adaptive for reuniting with absent partners; maladaptive when the partner is dead	humans, mammals, and birds forming attachment bonds	[11,89,90]
	incentive disengagement	grief co-opts foraging frustration mechanisms to break attachment to a social partner who no longer provides reward	highly social mammals; absent in solitary basal mammals	[91]
	reunion vigilance	preoccupation maintains low detection threshold for absent partner; false recognitions decline as cognitive representations update	humans, mammals, and birds forming attachment bonds	[92]
**functional (cognitive)**	searching–waiting strategy	protest and despair are poles of a single adaptive strategy; the organism switches by cost–benefit appraisal, conserving energy (waiting) when searching cannot resolve the loss	mammals facing unsolvable survival problems	[39]
	prevent future loss	grief as specialized sadness, a partially differentiated subtype of a generic negative-affect system. Pain of loss instils lessons to avoid circumstances that led to loss; root cause analysis identifies preventable causes	humans and species capable of causal learning	[93–95]
	update cognitive map	reduced activity allows updating internal model across hundreds of schemas representing the lost individual	humans and species with complex cognitive maps	[96]
**functional (social)**	signal commitment	hard-to-fake signal of bond-forming capacity to prospective social partners	humans and social species with free partner choice and non-kin bonds	[97,98]
	solicit help/bargaining	costly signal of genuine need to existing social partners enmeshed in conflict	humans and social species with interdependent relationships	[99,100]
	ensure group cohesion	grief makes separation punishing, reinforcing social bonds necessary for survival in individualized groups	humans and species forming individualized groups based on personal bonds	[101]
**integrative/mechanistic**	panic→seeking suppression	protest = PANIC/GRIEF + SEEKING active; despair = PANIC still active, SEEKING suppressed by neurochemical changes	humans and mammals; homologous circuitry in birds	[102,103]
	cytokine-mediated sickness behaviour	proinflammatory cytokines mediate transition from active protest to passive despair; inflammatory dysregulation may escalate adaptive response to pathological depression	humans and mammals	[31,104]
	sadness as cue, grief as signal	sadness is a self-directed low-arousal ethological cue (cognitive processing); weeping is an other-directed high-arousal ethological signal (soliciting help), ritualized from immune-mediated allergic symptoms	sadness: humans and mammals; weeping/tears: humans	[105]

### (a) Grief, an emergent property of ancient caring pathways?

Many of the evolutionary hypotheses reviewed above have a similar shortcoming: they describe the functions or consequences but do not explain why it emerges so broadly across vertebrates. Some ground the grief response in phylogenetically ancient attachment/separation distress systems. Such bonds are mediated by neuropeptide hormones such as OT (in mammals) and its homologues, such as mesotocin (in birds, reptiles and fishes). Among vertebrates, these hormones originally served basal reproductive behaviour (vocalizations, courtship behaviours and nest building) and were then extended towards pair bonding, parental care and the formation and maintenance of social bonds (reviewed in [106]). The recruitment of OT-like peptides into social bonding required numerous anatomical developments. In basal vertebrates, magnocellular neurons reside in a single *preoptic nucleus* and discharge peptide primarily into the cerebrospinal fluid, mediating slow, stereotyped reproductive behaviours [106,107].

In amniotes, the *preoptic nucleus* diverges into the *paraventricular* and *supraoptic nuclei*, and axonal projections expand into forebrain regions including the NAc, *central amygdala* and *lateral septum*. This expansion permits rapid, targeted release of neuropeptide into circuits governing selective social bonds. In birds, mesotocin in the *lateral septum* mediates preference for familiar conspecifics [108]. In mammals, OT in the NAc is required for the formation and maintenance of pair bonds [109].

Pair bonding itself is not homologous across these taxa. It evolved independently many times (e.g. prairie voles and zebra finches did not inherit their monogamy from a common pair-bonding ancestor). However, because independent transitions to pair bonding convergently recruit the same homologous nonapeptide toolkit [67], the disruption of that toolkit upon loss produces functionally equivalent distress across species.

**Figure 2.**
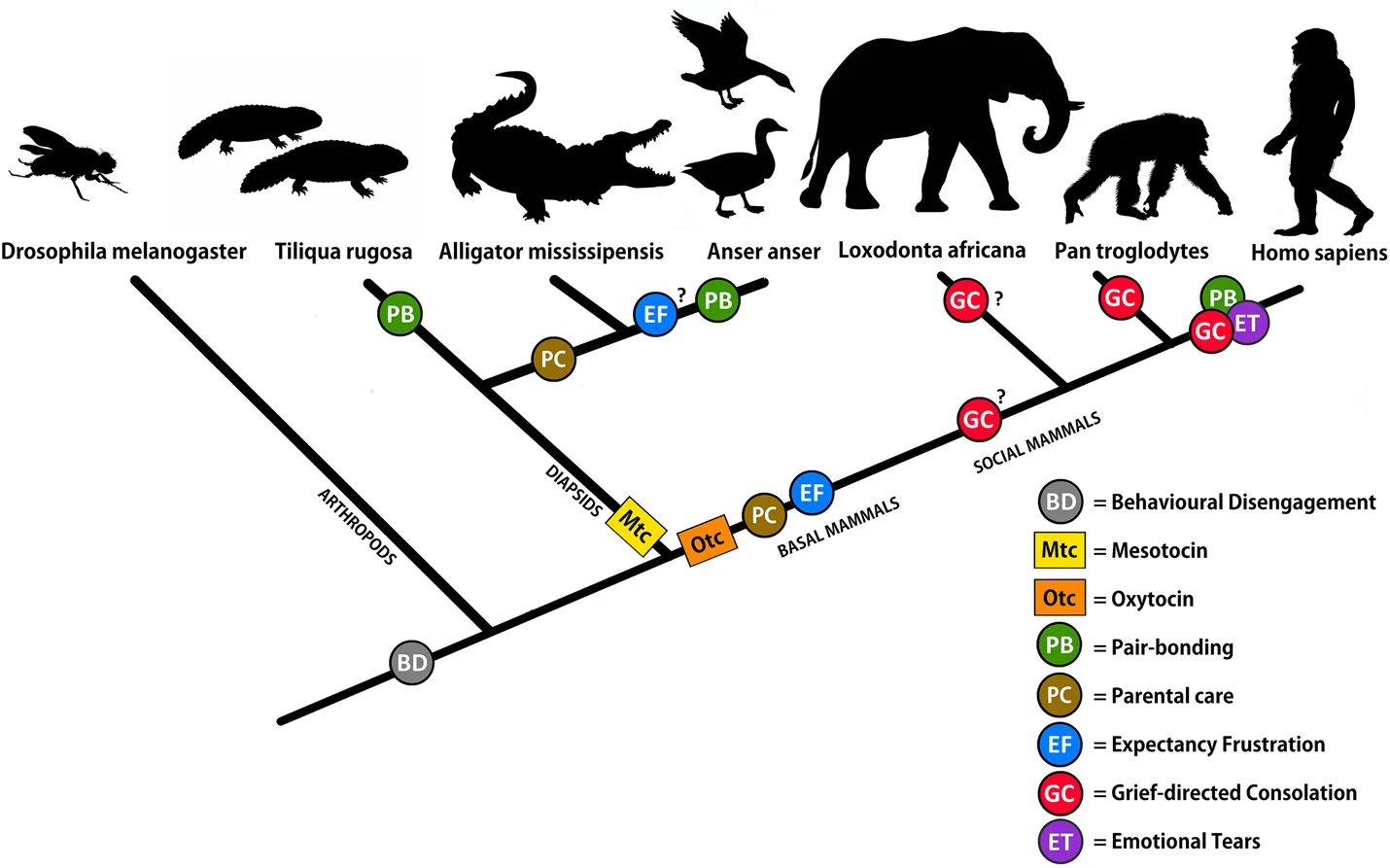
Evolutionary emergence of grief-related responses and associated neuroendocrine systems. Schematic cladogram showing the proposed distribution of social-attachment and grief-related traits across selected taxa. Coloured nodes indicate the inferred evolutionary origin of each trait along the corresponding lineage. ‘Social mammals’ denotes a behavioural grouping rather than a monophyletic clade. Question marks indicate uncertainty in the phylogenetic placement or evolutionary origin of a trait.

Grief could then be thought of as an emergent consequence of a deep homology. In all cases, because the toolkit is shared, the disruption of any bond it mediates produces functionally equivalent distress. Below, we propose an integrated evolutionary account for how grief might have emerged, and why it took the shape it currently has in some lineages, including humans. We add a cladogram tracing major transitions in that evolutionary trajectory (figure 2).

#### (i) Layer 1. Behavioural disengagement: the ancient intrapersonal substrate

When an organism encounters circumstances it cannot escape or remedy, continued effort wastes metabolic resources and may expose the organism to further harm; selection favours mechanisms that detect uncontrollability and modulate behaviour accordingly. The phenomenon is widely distributed and substantially conserved. *Learned helplessness* (i.e. failure to escape escapable shock following exposure to inescapable shock, reversion to non-escape behaviour after successful escape, passivity under escapable shock after inescapable training) has been demonstrated across phyla, including in invertebrates that diverged from the vertebrate lineage more than 600 million years ago [110]. The basic paradigm was established in cockroaches and locusts [111]. A study in fruit flies demonstrated the same phenomenon with two separable components: a cognitive suppression of futile escape responses and a motivational downregulation of locomotor activity that the authors describe as ‘possibly mood-like’ [112]. Subsequent research confirmed that these effects stem from uncontrollability, not the stressor, and are reversible: yoked flies realign once they regain control [113].

Taken together, this body of work supports framing *behavioural disengagement* at the deepest layer as an ancient and adaptive regulatory response. In the wild, the response is adaptive: becoming quiescent in the presence of an uncontrollable threat reduces energy expenditure and, in animals with motion-detecting predators, can blend the organism into the background until the threat passes [111]. This layer establishes only that an internal state of regulated disengagement under uncontrollable adversity exists, is ancient and adaptive and, arguably, the substrate on which grief is built.

Within mammals specifically, this disengagement substrate appears to have been elaborated into a more specific mechanism. Papini and colleagues [91] propose that *frustrative non-reward* (the behavioural response to reward devaluation or omission) evolved in Mesozoic mammals or their cynodont ancestors as an *incentive disengagement mechanism*, possibly co-opted from earlier fear circuitry. Its adaptive function is to break attachment to a site or stimulus that has stopped providing rewards and to invigorate search for alternative sources, an adaptation responding to the high energy demands of endothermic, large-brained, dynamically active mammals. *Frustrative non-reward* shows a different phylogenetic distribution than the broader behavioural-inhibition substrate. The downshift produces frustration (an aversive emotional reaction to the loss, coded behaviourally and supported by the response to anxiolytic drugs) that goes beyond simple recalibration to the new reward value. Importantly, Papini and colleagues [91] propose that this *incentive disengagement mechanism* was secondarily co-opted in highly social mammals from its original foraging context into the realm of social loss. Grief, on this account, is *incentive disengagement* applied to bonded individuals (social reward) rather than to foraging sites (food reward). The mechanism that breaks attachment to a devalued food source can be repurposed to break attachment to a lost bonded partner, freeing the animal to resume other vital functions.

#### (ii) Layer 2. Attachment-loss response: dyadic distress signalling in vertebrate bonds

The distress vocalization is one of the most deeply conserved features of vertebrate behaviour. Across mammals, birds and crocodilians—taxa whose lineages diverged from each other more than 300 million years ago—the acoustic structure of infant separation calls is fundamentally similar. Moreover, the neurochemical systems modulating these calls involving opioids and OT are the same systems that mediate adult social bonding [114]. On the receiver side, infant distress calls induce cortisol elevation and physiological arousal in carers, motivating them to respond in order to terminate the aversive stimulus [115]. They use the same conserved neurochemicals at different stages of development, which is what allows adult grief to be understood as a co-option of the infant separation distress system using shared circuitry [102]. Within this broadly conserved framework, Newman [116] described the *mammalian cry circuit*, encompassing both infant production and carer response, as a system that arose early in mammalian evolution as an adaptation for maintaining mother-infant contact and has been broadly conserved across the eutherian lineages studied.

This same vocalization-and-bonding system underlies two kinds of bonded relationships across vertebrates: the mother-infant dyad and the pair-bond. Both produce distress vocalizations and search behaviour when the partner is absent. The first is the mother-infant dyad. Early psychologists framed infant distress vocalization as an attachment behaviour whose function is maintenance of proximity between dependent infant and carer [89,90]. The selective rationale operates within a sender-receiver framework in which infant and mother share substantial genetic affinity, and crying functions both to recruit care and to convey information about the infant's condition [117]. A crucial point is that the vocalization does not replace the underlying disengagement programme from layer 1 but rather overlays it. When the signal works (i.e. the mother comes, proximity is restored), the disengagement programme is averted. When the signal fails (i.e. separation is prolonged, reunion does not occur), the underlying programme asserts itself and the infant transitions from active vocalization to passive withdrawal. Bowlby and colleagues documented this *protest-despair sequence* in maternally separated human infants in the 1950s, and Harlow and colleagues replicated it experimentally in rhesus macaques.

The second dyadic structure is the pair-bond. As established above, pair-bonding evolved convergently across vertebrate lineages, but the underlying nonapeptide toolkit is conserved and continuous with the homologous mother-infant attachment system that runs across amniote tetrapods [67,118]. The vocalization-and-search response to bond disruption looks the same in both kinds of dyad because it runs on the same conserved circuitry, whether the bond was inherited from a common ancestor or evolved convergently. Pair-bonded birds give extended contact calls and search vocalizations when they lose a partner; pair-bonded mammals do similarly, and that loss produces the same increase in vocalization and searching underpinned by neuroendocrine changes [51].

#### (iii) Layer 3. Grief-directed consolation: a cue-to-signal transition?

The internal disengagement state and the mother-infant distress vocalization system established in earlier layers are joined by a broader repertoire of visible *behavioural* and *postural cues* that conspecifics can in principle read. The mother-infant signalling system, like the pair-bond, evolved within a dyadic communication channel: the infant's distress vocalizations were under selection to be read by the mother, and her caring responses were under selection to be deployed towards her infant. For bereavement-related distress to become visible to anyone beyond that dyadic partner, the bereaved must exist within a wider social structure in which non-dyadic conspecifics are in a position to detect the cues. For example, hunching, slumped posture, self-clutching, reduced locomotion and withdrawal from social engagement are documented across primates in contexts of maternal loss and social separation. The facial musculature for producing distress configurations is present and homologous across great apes, although no species-typical grief expression has yet been documented in any non-human species [119]. Observations consistent with active distress, including vocalization and attempts to revive dying conspecifics and temporal gland streaming at carcasses [120,121] and with passive behavioural withdrawal in bereaved survivors [122], have also been documented in elephants.

An important point is that these visible cues remain cues in the technical sense, rather than signals [123]. In this sense, a cue conveys information that benefits the receiver but is not itself shaped by selection on the sender, while a signal is shaped by selection on the sender because receivers respond in ways that benefit the sender. The transition from cue to signal requires that receiver responses become reliable and beneficial enough to drive sender-side elaboration. The comparative primate evidence shows that receiver responses to distress responses exist. Chimpanzee consolation behaviour is well documented [124], and chimpanzee responses to bereavement (including extended carrying of infant corpses) have been described in multiple populations [125]. However, these responses are concentrated among close affiliates and are not widespread across primate species. Presumably, the default ancestral response in animals to visible weakness in a conspecific is opportunism: predators exploit visible distress, competitors exploit visible weakness [126]. Fessler & Moya [127] argue that even within-species adult responses to crying infants can include aggression and infanticide rather than care. As such, mixed and unreliable receiver responses generate weak sender-side selection, which is perhaps why the full repertoire of distress cues (i.e. postural, behavioural and vocal) remains observed primarily within the mother-infant signalling system rather than functioning as adult-to-adult signals in the broader social group.

The contrast between chimpanzees and other systematically studied primates becomes more interesting in the context of the broader empathy literature. Consolation (i.e. an affiliative response directed at a distressed conspecific, with at least some evidence for an emotional reaction in the observer) has been documented across a wide taxonomic range: great apes, several macaque species, canids, elephants, cetaceans, rodents and corvids [128]. Almost all of this evidence, however, concerns consolation in non-bereavement contexts: post-aggression affiliation, comforting of stressed or fearful conspecifics, prosocial response to bonded partners in distress. The deployment of these consolation behaviours into a bereavement context (grief-directed consolation), was documented under controlled observations only in chimpanzees [75], but scattered anecdotal cases in other primates hint at a broader capacity.

#### (iv) Layer 4. Grief ritualization: the sadness facial display and emotional tears

A fourth layer concerns hominins, whose evolved receiver ecology shifted the expression of vulnerability from concealment to display. It was a transition from cue to signal, from infant-directed to adult-to-adult communication and from within-kin to across-group signalling. It appears to be a hominin-specific evolution from the late Pliocene/early Pleistocene onwards. Rather than just a disposition to console, what hominins added was the apparatus to recruit consolation under a receiver ecology that made it safe to advertise vulnerability instead of suppressing it. Kret *et al.* [119] note that the existing distress repertoire in non-human great apes appears adequate to elicit consolation in receivers without the need for further elaborated signals. This may explain why ritualization did not occur in non-human apes despite the affective and anatomical substrate being in place. There may have been two convergent changes, one on the receiver side and one on the sender side, that account for the later hominin elaboration.

The first was a receiver-side change. The *cooperative-breeding hypothesis* [129,130] traces this shift in the elaboration of alloparental care for dependent infants. Hominin infants became too costly to raise on maternal investment alone, and a social ecology developed in which non-kin adults reliably responded to infant distress with prosocial behaviour. This suggests that the adoption of cooperative breeding by hominin ancestors is the most parsimonious account for human hyper-cooperation. Another, the *self-domestication hypothesis,* finds a partially overlapping change elsewhere [131]. During the late Pleistocene, selection against reactive aggression drove this change, with prosocial responsiveness emerging as a correlated by-product of broader domestication traits. The two hypotheses differ in timing and in proximate mechanism (cooperative breeding plausibly originated in early *Homo*, self-domestication probably in *Homo sapiens*), but both predict the same crucial outcome. That is, receiver-side responses to distress displays became more reliable and prosocial enough, across the broader social group rather than only among close kin, to drive sender-side selection on the display.

The second was a sender-side change. The involuntary facial musculature of distress, previously available only as a cue, became ritualized in the ethological sense (i.e. exaggerated, stereotyped, more visible and more reliably produced under appropriate conditions). The sadness facial configuration that Darwin originally described, with the obliquely raised inner eyebrows and the downturned corners of the mouth, became the species-typical grief display. Tears were co-opted as part of this ritualization. The by-product of strong infant eye-muscle contractions, which had been available within the mother-infant system, was extended into the adult distress signalling repertoire. Gračanin and colleagues' [132] analysis of the three functions emotional tears serve once established (soliciting succour and care, promoting social bonding and inhibiting aggression) describes the selective pay-offs that would have favoured this extension. The aggression-inhibition function in particular, supported empirically by Riem *et al.* [133], gives tears a function that vocal crying cannot perform: lacrimation blurs vision and degrades the crier's capacity to fight or flee, positioning tears as a handicap and a reliable signal of appeasement, need, or attachment that cannot be cheaply faked [134]. Adult tears track helplessness, loss, separation and attachment-related events rather than physical pain [135]. This is consistent with a bonding-based origin: tears recruit help in contexts of disrupted bonding, loss and separation, rather than in contexts of physical pain or injury, where concealment of vulnerability would have been advantageous.

Darwin held that the tears and facial contortion of grief first arose to shield the eyes during the violent expirations of infant screaming (a protective reflex, incidental rather than communicative), later carried into adult emotional expression where that original function is no longer primary [9]. Bylsma *et al.* [136] note that, unlike the basal and reflex tearing common to mammals, the shedding of emotional tears appears to be uniquely human. They propose a possible link to von Economo neurons of the *anterior cingulate* and *frontoinsular cortex* (regions central to interoception and the affective dimension of emotion). The link to emotional tears is so far conjectural, resting largely on the observation that both von Economo neurons and emotional tearing are diminished in familial dysautonomia.

Ultimately, the biological signalling system established in the *Homo* lineage became the substrate for cultural elaboration. Ritual weeping, the codification of display rules governing when and how grief may be shown, the gendered patterning of public weeping documented across societies and the religious uses of tears all operate on this evolved substrate but extend it through cultural transmission [132]. While grief is evolutionarily ancient and widely shared across taxa, what appears distinctively hominid is its translation into formalized action, identifiable archaeologically through deliberate burial in socially meaningful places. Such burials, in camps and shelters used by the living, emerge in the Middle Palaeolithic around 120 000 years ago among both Neanderthals and early anatomically modern humans. They mark what some would describe as an early ritualized bridge between the living and the dead, suggesting that social bonds could extend beyond death through continued care and attention [137]. Emotional tears, on this view, are one signal within a grief-and-affiliation system whose affective core is shared across social mammals but whose ritual forms may be a uniquely hominid inheritance.

## 5. Conclusion

Throughout this review, we assembled the comparative case for grief from several converging traditions that rarely interact: the comparative study of its facial expression from Darwin onwards, the psychology and neurobiology of separation and pair-bond disruption, the ethology of bereavement in free-ranging primates, the owner-report literature on companion animals and evolutionary accounts of depression and sadness together with their underlying mechanistic pathways. Drawing these together, we proposed a layered evolutionary scenario for grief: (i) an ancient, broadly conserved substrate of behavioural disengagement under uncontrollable adversity; (ii) a dyadic distress-signalling system shared between the mother-infant bond and the pair-bond; (iii) the emergence of grief-directed consolation where receivers respond reliably enough to distress; and, in the hominin lineage, (iv) the ritualization of the sadness display and emotional tears into a grief signal following a change in social ecology. It remains a scenario rather than a settled account, and the most consequential gap concerns the consolatory response of conspecifics to bereaved individuals.

Whether and under what conditions non-human animals console the bereaved is one of the most empirically open questions in the field. Group-living animals have not been reliably observed consoling bereaved members, though the Goldsborough *et al.* [75] chimpanzee study offers preliminary evidence for grief-directed consolation in great apes. In these conditions, we do not know which species console, under what circumstances, or in response to which cues. Nor do we know whether a bereaved individual is recognized through facial expression, posture, vocalization, behavioural change, olfactory cue or the simple absence of the partner.

We have made the integrative case for why grief emerged among distinct taxa. Our views rest on integrating many separate literatures into a coherent narrative. The most we can claim is to have offered a general framework in which these questions can be productively asked, and predictions that the coming generation of comparative thanatology may be equipped to test.

## Supplementary Material



## Data Availability

Supplementary material is available online [138].

## Theme

One contribution of 19 to a theme issue ‘Mechanisms, development, phylogeny and functions of emotional expressions’.
