# Supplementary material for: On the nature of grief: uncovering the evolutionary roots of loss across the animal realm
Source: Philos Trans R Soc Lond B Biol Sci. 2026 Sep 24;381(1959):20250147. doi: 10.1098/rstb.2025.0147 (PMC13601878; doi:10.1098/rstb.2025.0147)
Supplement: Supplementary file 1 [file rstb.2025.0147.zip › rstb.2025.0147/rstb20250147_si_001.pdf]

Supplementary Data:
On the Nature of Grief: Uncovering the Evolutionary Roots of Loss Across the Animal Realm

Sarv Dashti[1] & André Gonçalves[1,2]

1 Center for the Evolutionary Origins of Human Behaviour, Kyoto University Japan.
2 Science and Research Department, Indianapolis Zoological Society, USA.

1. The nature of grief: theoretical and comparative foundations

(a) Cultural variation and Biological universals

What is meant when we invoke terms such as *grief*, *mourning*, or *bereavement*? Often, in the human literature, these concepts are vague or confused with each other. Here we draw on the more inclusive conceptualisations of grief offered by authors who included non-human animal perspectives (Parkes, 1972; Averill, 1979; Archer, 1999). Bereavement is the objective status or period during which an individual faces a loss due to the death of a bonded individual; it has a temporal aspect during which grief is experienced and mourning is manifested. It is a dynamic, universal, and yet individualised process with a normative component. Grief is characterised as the emotional, physiological, cognitive, or behavioural responses towards the loss of a conspecific, companion, or even an object. This response may be triggered by physical separation (perceived loss) or death (actual loss). Mourning refers to the observable behavioural manifestations of grief, which, in the case of humans, are typically ritualised and shaped by social and cultural factors such as death rituals and practices that serve a coping function.

A clearer distinction can thus be drawn between mourning and its related terms. Grief is better understood not as a single basic emotion, but as a prolonged state within which a constellation of emotions and motivational states (sadness, anger, guilt, anxiety and yearning), may arise. Mourning is culturally mediated and therefore variable across human societies, while grief itself appears to have a universal biological substrate. The strongest evidence for this comes from cross-cultural research on bereavement and mortuary practices. Rosenblatt, Walsh, and Jackson (1976), in one of the most comprehensive cross-cultural studies of bereavement to date, examined ethnographic data from 78 world cultures and concluded that emotional responses to bereavement (including crying, overt anger, and fear) constitute a basic human characteristic, present across the vast majority of societies studied.

At the same time, the cultural shaping of how, when, and for how long these responses are expressed varies enormously. Some societies institutionalise anger and aggression in bereavement, others suppress emotional expression almost entirely, and mourning duration, remarriage rules, and ceremonial structures differ dramatically across cultures (Rosenblatt et al., 1976; Parkes, 2001). Traditionally, the Navajo limit mourning to four days, after which any expression of grief or mention of the deceased is prohibited; the Balinese smile through bereavement in the belief that displaying distress would harm others; among the Yoruba of Nigeria, dead infants were sometimes disposed in remote locations to avoid offending earth shrines; the Samoans reported that typical Western signs of grief were unknown to them; and the Tiwi of Australia practise self-mutilation in mourning, understanding the deceased as the loss of part of one's own body (reviewed in Stroebe & Schut, 1998). Among the Yanomami of Venezuela, Eibl-Eibesfeldt and Mattei-Müller (1990) recorded dawn wailing songs in which bereaved parents mourned a dead child often through the imagery of their shared daily activities, lamenting the fishing and foraging excursions they would never take again. Yet beneath this variation, grief remains in essence a universal human response to loss especially

the loss of a significant person (Stroebe & Schut, 1998), and certain structural features of mortuary practice are remarkably consistent. Rosenblatt et al. (1976) found that most cultures prescribed the destruction of a deceased person's property (91%), abandonment of its residence (81%), and impose a taboo on naming the dead (59%). They also found a positive association between the absence of a final ceremony and prolonged grief, measured by disrupted work, troubled dreams, suicidal behaviour, and illness, suggesting that mourning rituals functionally reduce the duration of grief-induced impairment (Rosenblatt et al., 1976).

Extending on these findings, White, Marin, and Fessler (2017) conducted the most extensive systematic cross-cultural investigation of corpse interaction in mortuary rituals, examining ethnographic records from 57 representative cultures. About 93% involved visual exposure of the corpse to kin, 89.5% involved physical contact and 82% of that contact was moderately intimate (i.e. washing, dressing, kissing, and embracing the body). Highly invasive practices (dismemberment, consumption of remains) were rare, occurring in only 8% of cultures. They found no regional differences in the level of intimacy. Because the sampling controls for shared cultural history, the authors conclude this near-universal pattern likely reflects convergent cultural evolutional rather than historical diffusion. White and colleagues (White et al., 2017: White & Fessler, 2018) proposed an evolutionary account for this convergence. Drawing on both the *reunion theory of grief* (Bowlby, 1982), which holds that grief's primary domain is the search for reunion with a lost partner, and the *reorientation theory* (Nesse, 2005), which posits that grief facilitates disengagement from the deceased and reorientation toward new relationships, they argue that grief deploys both mechanisms in sequence. The initial phase is dominated by reunion-seeking behaviours: yearning, searching, and vocalisation. As evidence accumulates that the valued other is dead, the process shifts toward cognitive recategorisation of the deceased from living relationship partner to non-viable partner, opening the door to replacement relationships. The corpse is the most reliable cue of death, and as such, plays a critical role in this transition. Prolonged visual and tactile contact with an immobile, cold, unresponsive body provides the compelling sensory evidence needed to overcome the bereaved individual's unconscious scepticism regarding the death. In this scenario, an error-management bias toward continued searching takes place because prematurely abandoning a valuable individual is costlier than continuing to search (White & Fessler, 2018; White et al., 2017). Touching corpses in a moderately intimate way (82% of cultures) seems the best compromise between the benefits of seeing death-cue and the costs of triggering evolved disgust mechanisms that protect against disease risk (White et al., 2017, see also Tybur et al., 2013: Sarabian et al., 2026).

This framework may have an important phylogenetic dimension as rudimentary urges to engage in tactile contact with corpses have been documented in social birds and mammals. Naturalistic observations frequently report mothers often carrying dead infants, touching them, and sometimes letting other group members handling them before abandoning the body some time later (Anderson, 2016; Gonçalves & Biro, 2018).

(b) Bowlby & Parkes: A Biological Theory of Grief

Nearly a century later, Darwin's observations lay largely unacknowledged in the grief literature until the late 1950s. Colin Murray parkes recalled that as he began reading the available scientific literature available on loss he was struck by the near-total absence of the observation that animals exhibited many of the features found in humans (Parkes, 1972; 2001). Reading Darwin, Parkes developed his "biological theory of grief", which remained influential over the following decades (Parkes, 1972). Not long after, he discovered that John Bowlby had independently reached many of the same conclusions. Their subsequent collaboration produced one of the most influential frameworks in bereavement research.

Bowlby's attachment theory (1969; 1973) provided the developmental and evolutionary support for grief responses. Infants develop specific behaviours from birth (i.e. crying, smiling, clinging, following) that work together with maternal caregiving to form early attachments. These behaviours were not uniquely human with other animals sharing the same or functionally similar ties to their progenitors (Parkes, 1972). The infant

uses the mother as what Parkes termed a "mobile base". With her, the infant explores the world, and retreats when something unfamiliar/threatening appears. When the attachment figure is near, novelties are approached. As the infant matures, it acquires the ability to tolerate greater absences, along with forming attachments to other individuals but the desire to maintain a safe familiar environment persists throughout life. When these bonds are severed in adulthood, the same behavioral systems established in infancy are activated. Working in parallel with Bowlby, Parkes undertook the first longitudinal study of widows through their first year of bereavement. Parker's (1970) studies showed psychological changes which resembled the sequence that Robertson & Bowlby observed in separated children (Robertson & Bowlby, 1952). The Bowlby and Parkes duo (1970) published a descriptive classification on the variable phases of grief which included: a) *numbness*, b) *yearning and searching*, c) *disorganisation and despair*, and d) *reorganisation*, recognising that individuals move back and forth rather than following a fixed sequence.

The central theoretical insight was Parkes's reconceptualisation of grief as searching behaviour which was found across animal species. Drawing from Darwin alongside ethologists like Lorenz and Tinbergen, he argued that the main characteristics of grief were not only the prolonged depressive periods but also the acute episodic "pangs" (i.e. waves of severe anxiety and psychological pain during which the lost person is intensely missed) (Parkes, 1972). He proposed that "pining" (the persistent, obtrusive wish for the person who is gone) is the subjective and emotional component of the urge to search for a lost object, and that an adult human being retains the same impulse to search that is shown by many species of social animal (Parkes, 1972). Parkes cited Lorenz's description of greylag geese separated from its mate (restless movement by day and night, long-distance calling, extended searching expeditions) and Bowlby's review of bereavement responses in jackdaws, geese, dogs, orangutans, and chimpanzees (Bowlby in Parkes, 1972). He also drew on primate studies of vervet monkey "lost calls" (Struhsaker, 1967). Struhsaker's spectrographic analysis revealed them to be penetrating and capable of accurate directional location over long distances. The survival value of such behaviour was clear: crying and searching made it more likely that the lost individual would be recovered. Moreover, the separated individual must be prepared to fight or flee, hence the importance of an alarm reaction during the early and more active phase of grief.

Parkes extended Darwin's insight about the facial expression of grief into a broader social-signalling framework. The suppressed cry of the grieving adult (with the raised inner eyebrows, wrinkled forehead, depressed mouth corners), has become part of a ritualised social signal system and under little volitional control but which enabled "the separated individual to evoke and direct the help of others in an orderly and constructive manner" (Parkes, 1972, p.43). It was only in the tragic but statistically infrequent event of permanent loss this involuntary expression of grief lose its utility. Although the bereaved adult is typically aware that searching for a dead person is irrational, the impulse persists. This was what Parkes, drawing on the ethological terminology, characterised as the frustration of appetitive behaviour.

The Bowlby-Parkes synthesis thus established grief as a biologically grounded response with deep evolutionary roots: an attachment system whose costs in the event of permanent loss are the price of its benefits across the rest of life. Their framing did in part answer but still left open questions that subsequent evolutionary and comparative accounts would take up in distinct ways. If grief is the by-product of an adaptive attachment system rather than an adaptation in its own right, what, if anything, does its persistence and severity in some individuals tell us about its function?

2. Companion animal grief: public attitudes, owner reports, and veterinary perspectives

Death, absence, and methodological considerations

There is also the question of whether animals differentiate between a companion's death and mere absence. The practice of allowing surviving animals to spend time with the body of a deceased companion

has been maintained by a few organisations, from zoos and sanctuaries to veterinary clinics and rescue groups (King, 2013). For instance, members of the House Rabbit Society, a US-based rescue organisation, routinely place the body of a deceased rabbit with the surviving partner for "viewing" (Smith, 2005). They report that surviving rabbits typically investigate the body, grooming it and lying beside it as they would a living partner, before gradually withdrawing. The current evidence is mixed with studies reporting no behavioural differences after viewing the body of a companion (Morris et al., 2016; Uccheddu et al., 2022) while others do (Ricci-Bonot et al., 2025, Dickinson & Hoffmann, 2016; 2019).

A survey of general practice veterinarians in South Carolina, USA (N=153; 70 women, 83 men) conducted by Dickinson and Hoffmann (2016) found that 54% reported observing behavioural changes in nearby animals at the time of euthanasia. The most common change was a sudden silence, followed by unusual sounds. Dogs (63%) and horses (8%) were most often mentioned. Changes were more common among animals from the same household than among unrelated individuals. However, these changes were not consistent across euthanasia events, and the veterinarians offered competing explanations: animal grief and empathy (24%), responding to humans' emotional cues (20%), responding to changes in the physical state of the dying animal (14%), pack disruption (9%), and olfactory detection of death (8%). Official guidelines from the American Veterinary Medical Association recommend against euthanising in the presence of other animals. Nevertheless, a survey subsequently showed that hospice-specialised veterinarians (N=137; 126 women, 11 men), carrying out on average twice as many euthanasias per year, actively prefer other animals to be present and find that survivors who witnessed a death tend to search for their dead companion for a shorter period (Dickinson & Hoffman, 2019).

Despite the challenges of anthropomorphism, pet owners' subjective reports on their animals' emotional states can be valuable for understanding grief in animals, particularly when, as Morris et al. (2008) demonstrated, those reports are grounded in specific, consistent behavioural episodes rather than global beliefs. If we were to take the studies in this section, we observe several points that correspond to each other. Behavioural changes after the death of another companion, in dogs, cats and horses, with broad similar patterns: decrease feeding and playing, increased attention-seeking and vocalisations and changes in sleeping. The relationship between the animals predicted the strength of the changes rather than the duration of time spent together, in all three species. Across surveys in the UK, Australian, Italy and the United States, the public have broadly similar views that animals grieve, although this attribution varies with species, owner experience, gender and age.

However, questionnaire studies can carry several methodological limitations. For instance, they all rely on retrospective owner reports, often reported months of even years after the event which introduces substantial memory bias. None includes pre-loss baseline behavioural data against which post-loss changes could be measured. The public attitude surveys (McGrath et al., 2013; Walker et al., 2014a, 2014b) and the general veterinary survey (Dickinson & Hoffmann, 2016) achieved roughly balanced gender samples. However, all of the owner-reported behavioural studies are heavily skewed toward women: 82% in Greene and Vonk (2024), 90% in Uccheddu et al. (2022), 91% in Walker et al. (2016), and 97% in Ricci-Bonot et al. (2025). Walker et al. (2014b) found that women are more likely to attribute complex emotions to animals, but also noted that men are more likely to deny their own grief experience, making it unclear whether the gender imbalance inflates reported rates of behavioural chance or balanced sampling supresses them. Either way, it limits the generalisability of the results.

There is the issue of pervasive self-selection bias. For instance, owners who noticed changes in their animals were more likely to respond than those who did not, inflating reported rates of behavioural change. Research has not yet determined the question of whether reported changes reflect the animal's response to loss or the owner's altered behaviour. Uccheddu et al. (2022) found that owner humanisation of pets did not predict reported dog behavioural changes, which the authors took as evidence against simple projection. Greene and Vonk (2024), however, found that caregiver attachment strength did predict reported cat behavioural changes. Nonetheless, whether attachment style reflects anthropomorphic bias or as a legitimate variable reflecting real differences in the human-animal relationship and its consequences for the

surviving animal remains an open question. To date, no study has yet assessed death versus temporary separation using controlled comparisons. Yet, the Ricci-Bonot et al. (2025) finding that horses showed additional behavioural changes beyond those seen during routine separation after witnessing deaths offers preliminary evidence that the two may differ.

3. Evolutionary Accounts of Grief and Depression

(a) Non-functional accounts: grief as a cost of attachment

An early evolutionary explanation account of grief treats it as a by-product of the attachment system. Bowlby (1969, 1973) and Parkes (1972) proposed that natural selection equipped mammals with a motivational system that maintains proximity between infant and caregiver. When the caregiver is absent, the infant protests (crying, searching, and agitation) to signal distress and elicit reunion. When protest fails, the infant enters a *despair phase* characterised by withdrawal, reduced activity, and behavioural shutdown. They viewed grief in adulthood as the same attachment system responding to the loss of any significant relationship partner. The *protest-despair response* was documented in contexts where reunion remained possible, and grief following death was understood as the attachment system misfiring in a context where reunion is impossible.

Archer (1999, 2001) made this case explicitly. For him, grief is a maladaptive side-effect of a separation response that evolved to recover absent-but-living partners. Selection cannot strip grief out without also stripping out the attachment system that generates it, because the fitness gains from maintaining close bonds far outweigh the rare cost of losing one to death. He pointed to evidence that grief increases mortality, raises cortisol, suppresses immune function, and reduces interest in food and sex, taken together as a clear fitness cost. Archer (2001) further argued that non-human primates, including chimpanzees, do not reach the cognitive level at which children come to distinguish death from temporary absence, usually around six or seven years of age. He interpreted chimpanzee and other primate mothers carrying dead infants for diminishing periods before eventually abandoning them as gradual extinction of the separation response, not a response to death. He also dismissed the idea that grief might function by eliciting sympathy. In non-human primates, he noted, behavioural signs of grief are typically met with indifference by social companions (Seyfarth & Cheney, 1996; but see Goldsborough et al., 2020).

Nesse challenged Archer on this reasoning. The same evidence that Archer cited as proof of grief's maladaptiveness could equally be cited against pain, fever, or the stress response. The conflation of a defence with the suffering it produces is, Nesse argued, “an error that is as prevalent as it is serious” (Nesse, 2000b, p.60). If one loses touch with a close friend who moved away, one misses them. Learning later that the friend has died, something new and far more intense reaction takes over, that is qualitatively different from the sadness of missing someone. If grief were only the attachment system registering someone is gone, the knowledge they are gone permanently should extinguish it. Some emotions behave this way: when learning that a jealousy-provoking partner has died jealousy disappears and is instantly replaced with grief. Given grief does not switch off this way suggests it may be a distinct response to the specific situation of permanent loss, not merely a misfiring of separation anxiety. However, this argument inadvertently limits grief to species capable of representing death as permanent. Such position sits uneasily with Nesse's own claims elsewhere that grief is rooted in a phylogenetically conserved negative-affect system shared across mammals (Nesse 2000a; 2005). And yet, it could be argued that a reunion would extinguish grief just as quickly as the news of death that ignited it. If so, would not an emotion that switches off the instant the bonded partner returns remain tied to the separation-and-reunion system, rather than standing apart as a dedicated response to permanence?

White and Fessler (2018), offered another non-functional account on grief focused on the specific cognitive component of vigilance. Drawing on *signal-detection theory*, they suggested that preoccupation with the deceased maintains a low threshold baseline for detecting the absent partner in the environment. Since the cost of failing to detect an absent partner when alive (a miss) is higher than the cost of mistakenly perceiving the partner’s presence when dead (a false alarm), the system errs towards continued vigilance.

This leads to the phenomenon of false recognitions (e.g. hearing the deceased's footsteps, or glimpsing their face in a crowd). In two studies of bereaved pet owners, they found that the best predictor of false recognitions was the frequency of viewing photographs of the deceased. This is consistent with the idea that realistic images can lead low-level perceptual systems to recognize them as continuous viable relationship partners. Additionally, they found that seeing a non-intact corpse significantly reduced false recognitions, whilst viewing an intact corpse did not. This demonstrates that only unambiguous cues of death are sufficient to raise the detection threshold. After death, that readiness serves no reunion, which makes grief in the context of permanent loss a by-product of an otherwise adaptive system.

(b) Functional accounts: grief as adaptive

Cognitive functions

Several hypotheses propose that grief serves the individual by improving future decision-making Nesse (2000a; 2005) argued that natural selection began with a generic negative affect state common to mammals and then differentiated into subtypes specialized for particular unfavourable situations, with grief being one. This partial differentiation explains the overlap between grief and depression. Purposeful behaviours after a loss can include searching for what was lost, taking, protective action, warning kin, signalling need for help, and adjusting one's life strategies. He also drew a distinction between exchange relationships, where a substitute partner is readily available, and commitment relationships, where trust is built over long time and cannot be replaced without comparably large investment. According to this framework grief intensity does not simply track genetic relatedness or attachment strength but the irreplaceability of the lost relationship. Keller and Nesse (2006) provided empirical support for this differentiation across three studies. They found that different precipitants predicted distinct symptom profiles. Death of a loved one specifically was linked with emotional pain, crying, and desire for social support, whereas failure at an important goal was associated with guilt, rumination, pessimism, and fatigue. These results suggest that depressive symptoms are situation-specific responses shaped by natural selection for distinct adaptive challenges.

Thierry et al. (1984) proposed their own functional interpretation of the *protest-despair sequence* they termed *searching-waiting strategy*. Following Kaufman and Rosenblum's (1967) application of the *conservation-withdrawal model* to maternal separation in monkeys, they treated the two phases as opposite ends of one adaptive strategy. An organism facing an unsolvable survival challenge must decide whether to search which is energetically-costly (but raises chances of reunion), or to wait and conserve resources (when reunion unlikely). The organism updates this estimate over time and switches between poles accordingly. On this account, the *despair* phase is not a breakdown of the separation response but its adaptive complement. If searching fails, conserving energy is the better choice. When that conservation becomes persistent, it will present as depressive disorder, a pathological extreme of an otherwise functional continuum.

Welling (2003) proposed the *cognitive map hypothesis* in which depression following loss provides a necessary "time out" to update internal cognitive representations of the environment. After any significant loss, the individual's existing cognitive maps become inaccurate, because habitual actions that relied on the lost resource or person will fail. The number of schemas requiring updating determines the duration of the depressive response. Post-loss depression can act as a pause to update one's internal maps. This hypothesis predicts that species with more elaborate cognitive structures should show longer and more complex grief-like responses. It shares similarities with White and Fessler's (2018) reunion account, but Welling treats the depressive response as functional because it enforces a needed period for cognitive reorganization rather than a byproduct.

Andrews et al. (2021) provided an empirical test of the *prevention and rumination hypothesis* applied specifically to grief. Using a questionnaire administered to 619 bereaved help-seekers, they measured two dimensions of grief-related rumination: root cause analysis (analysing the cause of the loss) and reinvestment analysis (analysing how to redirect effort meaningfully). Among loss types, child death prompted the most rumination: older participants engaged in less root-cause analysis but more for other kin (siblings, nieces/nephews, grandchildren). This pattern is consistent with inclusive fitness theory: as people age, they shift cognitive effort from preventing direct fitness losses to preventing indirect fitness losses.

Social functions

Another class of hypotheses proposes that grief serves the individual by changing the behaviour of others. Averill (1968) presented an early evolutionary explanation, arguing that grief is a biological reaction whose function is to ensure group cohesiveness in species where social bonds are necessary for survival. By making separation from the group extremely stressful, grief discourages individuals from straying and punishes the disruption of social bonds. He limited the idea to species with personalized social groups (based on individual recognition and attachment) distinguishing from aggregations (held together by ecological conditions) and anonymous groups (held together by social attraction). Although those theoretical details have been revised, his core insight, that grief is strongest in species with individualized social groups, remains a testable prediction.

Winegard et al. (2014) later developed a social account based in costly signalling. In their *signalling model of grief*, the response functions as a hard-to-fake signal of a person's propensity to form strong, non-utilitarian social bonds. Because grief is costly (i.e. disrupts daily life, reduces fitness, increases mortality risk) and difficult to simulate physiologically, it provides observers with reliable information about the griever's underlying capacity for commitment. Reynolds et al. (2015) tested this prediction experimentally in humans. They found across four studies that high grievers were rated as more admirable, loyal, and trustworthy, were expected to cooperate more in a prisoner's dilemma, and were preferentially chosen as trust-game partners. These effects were specific to trust-based relationships and were not driven by pity or a general halo effect. Reynolds et al. (2020) extended this framework by proposing that grief intensity should be calibrated by life history strategy. Slow life-history strategists who invest in fewer long-term relationships, should grieve more intensely, because losing a high-investment partner imposes greater fitness costs.

Hagen (2011) proposed a different costly signalling account. While Winegard argued grief signals bond-forming capacity to prospective social partners, Hagen argued depression and suicidality are credible signals of genuine need directed at current partners involved in conflict. It could apply to any species with interdependent relationships where one partner can impose costs on the other by withdrawing effort. Watson and Andrews (2002) developed the *social navigation hypothesis*, proposing that depression serves two functions: a social rumination function (analysing solutions to social problems) and a social motivation function (motivating social partners to help, since the costs of depressive symptoms are borne not only by the individual but also by those who depend on them).

A challenge to such *social signalling accounts* comes from the comparative literature, particularly pair-bonded species. Rantala et al. (2018) noted that prairie voles show grief-like changes following partner loss (Sun et al., 2014), even though they do not seem to evaluate social partners in a complex manner. If grief were shaped by social selection as a signal to prospective allies, it should not appear in species that do not evaluate others' grief responses. Rantala and colleagues concluded that grief is likely a byproduct of attachment. Though it may have taken on a signalling function in human social communication, making that signalling a spandrel: a feature co-opted for a novel use after arising as a side effect of other adaptive processes.

(c) Integrative and Mechanistic Accounts

The hypotheses reviewed above differ in whether they view grief as non-functional, cognitively functional, or socially functional, but they are not always mutually exclusive. As Durisko et al. (2015) argued for depression more broadly, both functioning adaptations and biological malfunctions can produce the same symptoms, and different adaptive functions may account for different components of the grief response in different individuals or at different times. Then we have White and Fessler (2018) arguing that grief is not a unitary phenomenon but a bundle of cognitive and affective components. They argue that real progress depends on parsing these since each may have been shaped by distinct selective pressures. Their reunion vigilance account explains one such component, the preoccupation with the deceased and tendency to falsely perceive them as present, and recasts it as a signal-detection problem.

Several authors have specified the proximate neural and immunological pathways underlying the grief response, providing a level of analysis that is compatible with either functional or non-functional

interpretations. Panksepp and Watt (2011; see also Watt & Panksepp, 2009) located the pain of depression in the interplay between two primary-process emotional systems. The first, PANIC/Grief system, starts in the *periaqueductal gray* (PAG) moves through medial diencephalic structures and reaches the *anterior cingulate cortex* (ACC); social loss switches it on, producing psychological pain. The second, SEEKING system, is built on mesolimbic dopamine circuitry and produces positive emotion and drives exploration keeping the animal engaging with its surroundings. On their account, the *protest phase* of grief is what happens when both fire at once. The *despair phase* sets in later, when sustained PANIC/GRIEF activation begins to attenuate SEEKING, and once SEEKING is suppressed, the animal sinks into the lassitude, apathy, and withdrawal of full depression. Wright and Panksepp (2011) demonstrated this experimentally in rats. Using chronic electrical stimulation of the dorsal PAG, they found a sustained suppression of positive-affect vocalisations (an index of SEEKING system activity) that persisted for at least 29 days after the final stimulation, alongside reduced exploration and heightened anxiety-like behaviour. Watt (2023) situates this circuit-level account within a wider framework by treating depression and sickness behaviours as related forms of a conserved behavioural shutdown and tying the PANIC/GRIEF circuitry to an immune pathway into the same transition.

Hennessy et al. (2009) proposed such complementary immunological pathway for the *protest-to-despair* transition, based on studies of guinea pig pups separated from their mothers. The initial active response (vocalisation, locomotion) gives way to a passive response (immobility, crouching) that resembles sickness behaviour. They found that proinflammatory cytokines (interleukin-6 and other inflammatory mediators) are elevated during the passive phase, and that the passive response shares features with the sickness behaviour induced by immune activation. Rantala et al. (2018) extended this argument, to humans. They argued that modern human environments conducive to achronic inflammatory dysregulation (from obesity, sedentary behaviour, poor diet) prevent the normal recovery of mood and turn short-term grief into persistent depression.

Recently, Papini et al. (2022) proposed the *incentive disengagement hypothesis*, arguing that the aversive emotional state triggered by unexpected reward omission evolved in Mesozoic mammals to break attachment to a foraging site that no longer yields resources. The underlying frustration circuitry, in turn, may have evolved from the older fear circuitry by gene co-option (Papini, 2003), with its opioid modulation characterised in earlier work on recovery from reward loss (Papini et al., 2006). In their view, grief represents the co-option (exaptation) of this foraging mechanism into the social domain: when a bonded partner dies, the same frustration circuitry that would detach a forager from a depleted food patch is activated to break the social attachment. Papini et al. proposed a three-stage phylogenetic sequence, from fear (Palaeozoic vertebrates, antipredatory function) through frustration and incentive disengagement (Mesozoic mammals, foraging function) to grief (social mammals, social loss function). Their account predicts grief (presumably in its active and passive character) should appear independently in lineages that evolved complex sociality, such as primates and cetaceans and potentially birds. They propose that *frustrative non-reward* (FNR) was co-opted to social contexts. Epistemic frustration, the aversive state generated when an expected resolution of uncertainty fails to materialize, shows structural parallels to reward-loss frustration and recruits overlapping dopaminergic circuitry (Bromberg-Martin and Hikosaka 2009). The parallel suggests that the same disengagement logic (i.e., persistence, revision, or abandonment when expectations fail) may operate on information-seeking applied to both foraging and social bonds. Finally, the co-option of resource disengagement circuitry into the social domain proposed by Papini et al. is very much in line with *incentive disengagement theory* (Klinger, 1975) and the resource conservation component of broader evolutionary models of depression (Nesse, 2000a; Allen & Badcock, 2006). Both similarly propose that low mood facilitates disengagement from unreachable goals.

In another account, Huron (2018) treats sadness and grief as complementary but functionally distinct states. Sadness is marked by diminished physiological arousal, withdrawal, and reflective thinking. In this view, it functions like an ethological cue: it conveys information about the individual's state incidentally rather than communicatively, and can be indistinguishable from tired/sleepy states. Grief, marked by high arousal, weeping, and vocalisation, resembles an ethological signal: it is multimodal (visual tears, auditory wailing, and olfactory pheromones in tears), conspicuous, difficult to fake, and directly changes observer behaviour. He proposed that weeping evolved from immune mediated allergic reactions through the gradual ritualization process. The watery eyes and nasal congestion produced by histamine

release during stress originally served as incidental cues of the individual's depleted state, and were subsequently amplified by natural selection into a communicative signal because both signaller and observer benefited from the interaction.

References

Allen, N B., & Badcock, P. B. (2006). Darwinian models of depression: A review of evolutionary accounts of mood and mood disorders. Progress in Neuro-Psychopharmacology and Biological Psychiatry, 30(5), 815-826.

Anderson, J. R. (2016). Comparative thanatology. Current Biology, 26(13), R553-R556.

Andrews, P. W., Altman, M., Sevcikova, M., & Cacciatore, J. (2021). An evolutionary approach to grief-related rumination: Construction and validation of the Bereavement Analytical Rumination Questionnaire. Evolution and Human Behavior, 42(5), 441-452.

Archer, J. (2001). Grief from an evolutionary perspective. In Handbook of Bereavement Research (Stroebe MS, Hansson RO, Stroebe W, Schut H, eds), pp 263–283. Washington, DC: American Psychological Association.

Archer, J. (1999). The nature of grief: The evolution and psychology of reactions to loss. Florence, KY, US: Taylor & Francis/Routledge.

Averill, J. R. (1968). Grief: its nature and significance. Psychological Bulletin, 70(6, Pt. 1), 721.

Averill, J. R. (1979). The functions of grief. In Emotions in personality and psychopathology (pp. 337-368). Boston, MA: Springer US.

Bowlby, J. (1969). Attachment and loss: Volume I: Attachment. The Hogarth Press and the Institute of Psycho-Analysis.

Bowlby, J. (1973). Attachment and loss, vol. II: Separation. Basic Books.

Bowlby, J. (1982). Attachment and loss: retrospect and prospect. American Journal of Orthopsychiatry, 52(4), 664.

Bromberg-Martin, E. S., & Hikosaka, O. (2009). Midbrain dopamine neurons signal preference for advance information about upcoming rewards. Neuron, 63(1), 119-126.

Dickinson & Hoffmann (2016). The difference between dead and away: An exploratory study of behavior change during companion animal euthanasia. Journal of Veterinary Behavior, 15, 61-65.

Dickinson & Hoffmann (2019). Animal hospice and palliative care: Veterinarians' experiences and preferred practices. Journal of Veterinary Behavior, 32, 57-61.

Durisko, Z., Mulsant, B. H., & Andrews, P. W. (2015). An adaptationist perspective on the etiology of depression. Journal of Affective Disorders, 172, 315-323.

Eibl-Eibesfeldt, I., & Mattei-Müller, M. C. (1990). Yanomami wailing songs and the question of parental attachment in traditional kin based societies. Anthropos, (H 4./6), 507-515.

Goldsborough, Z., Van Leeuwen, E. J., Kolff, K. W., de Waal, F. B., & Webb, C. E. (2020). Do chimpanzees (Pan troglodytes) console a bereaved mother? Primates, 61(1), 93–102.

Gonçalves, A., & Biro, D. (2018). Comparative thanatology, an integrative approach: exploring sensory/cognitive aspects of death recognition in vertebrates and invertebrates. Philosophical Transactions of the Royal Society B, 373(1754), 20170263.

Hagen, E. H. (2011). Evolutionary theories of depression: a critical review. The Canadian Journal of Psychiatry, 56(12), 716-726.

Hennessy, M. B., Schiml-Webb, P. A., & Deak, T. (2009). Separation, sickness, and depression: a new perspective on an old animal model. Current directions in psychological science, 18(4), 227-231.

Huron, D. (2018). On the functions of sadness and grief. In The function of emotions: When and why emotions help us (pp. 59-91). Cham: Springer International Publishing.

Kaufman, I. C., & Rosenblum, L. A. (1967). The reaction to separation in infant monkeys: anaclitic depression and conservation-withdrawal. Psychosomatic Medicine, 29, 648–675.

Keller, M. C., & Nesse, R. M. (2006). The evolutionary significance of depressive symptoms: different adverse situations lead to different depressive symptom patterns. Journal of Personality and Social Psychology, 91(2), 316.

King, B. J. (2013). How animals grieve. University of Chicago Press.

Klinger, E. (1975). Consequences of commitment to and disengagement from incentives. Psychological Review, 82(1), 1.

Nesse, R. M. (2000a). Is depression an adaptation? Archives of General Psychiatry, 57(1), 14–20.

Nesse, R. (2000b). Book review: The nature of grief: The evolution and psychology of reactions to loss. Evolution and Human Behavior, 21, 59–61.

Nesse, R. M. (2005). An evolutionary framework for understanding grief. In Spousal Bereavement in Late Life (pp. 195–226). Springer.

Panksepp, J., & Watt, D. (2011). Why does depression hurt? Ancestral primary-process separation-distress (PANIC/GRIEF) and diminished brain reward (SEEKING) processes in the genesis of depressive affect. Psychiatry: Interpersonal & Biological Processes, 74(1), 5-13.

Papini, M. R. (2003). Comparative psychology of surprising nonreward. Brain, Behavior and Evolution, 62(2), 83-95.

Papini, M. R., Guarino, S., Hagen, C., & Torres, C. (2022). Incentive disengagement and the adaptive significance of frustrative nonreward. Learning & Behavior, 50(3), 372-388.

Papini, M. R., Wood, M., Daniel, A. M., & Norris, J. N. (2006). Reward loss as psychological pain. International Journal of Psychology and Psychological Therapy, 6(2), 182-213.

Parkes, C. M. (1972). Studies of grief in adult life. London: Tavistock.

Parkes, C. M. (2001). A historical overview of the scientific study of bereavement. In Handbook of bereavement research: Consequences, coping, and care (pp. 25–45). American Psychological Association.

Rantala, M. J., Luoto, S., Krams, I., & Karlsson, H. (2018). Depression subtyping based on evolutionary psychiatry: Proximate mechanisms and ultimate functions. Brain, Behavior, and Immunity, 69, 603-617.

Reynolds, T., Boutwell, B., Shackelford, T. K., Weekes-Shackelford, V., Nedelec, J., Beaver, K., & Abed, M. G. (2020). Child mortality and parental grief: An evolutionary analysis. New Ideas in Psychology, 59, 100798.

Reynolds, T., Winegard, B. M., Baumeister, R. F., & Maner, J. K. (2015). The long goodbye: A test of grief as a social signal. Evolutionary Behavioral Sciences, 9(1), 20.

Robertson, J., & Bowlby, J. (1952). Responses of young children to separation from their mothers. Courrier du Centre International de l'Enfance, 2(66), 131-142.

Rosenblatt, P. C., Walsh, R. P., & Jackson, D. A. (1976). Grief and Mourning in Cross-Cultural Perspective. New Haven, CT: HRAF Press.

Sarabian, C., MacIntosh, A. J., Gonçalves, A., Kawai, N., & Adachi, I. (2026). Dissecting disgust and fear in chimpanzees: From facilitation to disruption of cognitive processes. iScience, 29(8).

Seyfarth, R., & Cheney, D. (1996). Inside the mind of a monkey. In Readings in Animal Cognition (eds M. Bekoff & D. Jamieson), pp. 337-343. Cambridge, MA: MIT Press.

Smith, J. A. (2005). "Viewing" the body: Toward a discourse of rabbit death. Worldviews, 9(2), 184-202.

Stroebe, M., & Schut, H. (1998). Culture and grief. Bereavement Care, 17(1), 7-11.

Struhsaker, T. T. (1967). Auditory communication among vervet monkeys. In S. A. Altmann (Ed.), Social communication among primates (pp. 281–324). Chicago: University of Chicago Press.

Sun, P., Smith, A. S., Lei, K., Liu, Y., & Wang, Z. (2014). Breaking bonds in male prairie vole: long-term effects on emotional and social behavior, physiology, and neurochemistry. Behavioural Brain Research, 265, 22-31.

Thierry, B., Steru, L., Chermat, R., & Simon, P. (1984). Searching-waiting strategy: A candidate for an evolutionary model of depression? Behavioral and Neural Biology, 41(2), 180-189.

Tybur, J. M., Lieberman, D., Kurzban, R., & DeScioli, P. (2013). Disgust: evolved function and structure. Psychological review, 120(1), 65.

Watt, D. F. (2023). The separation distress hypothesis of depression—an update and systematic review. Neuropsychoanalysis, 25(2), 103-159.

Watt, D. F., & Panksepp, J. (2009). Depression: An evolutionarily conserved mechanism to terminate separation distress? A review of aminergic, peptidergic, and neural network perspectives. Neuropsychoanalysis, 11(1), 7-51.

Welling, H. (2003). An evolutionary function of the depressive reaction: The cognitive map hypothesis. New Ideas in Psychology, 21(2), 147-156.

White, C., & Fessler, D. M. (2018). An evolutionary account of vigilance in grief. Evolution, Medicine, and Public Health, 2018(1), 34-42.

White, C., Marin, M., & Fessler, D. M. (2017). Not just dead meat: An evolutionary account of corpse treatment in mortuary rituals. Journal of Cognition and Culture, 17(1-2), 146-168.

Winegard, B. M., Reynolds, T., Baumeister, R. F., Winegard, B., & Maner, J. K. (2014). Grief functions as an honest indicator of commitment. Personality and Social Psychology Review, 18(2), 168-186.

Wright, J. S., & Panksepp, J. (2011). Toward affective circuit-based preclinical models of depression: sensitizing dorsal PAG arousal leads to sustained suppression of positive affect in rats. Neuroscience & Biobehavioral Reviews, 35(9), 1902-1915.
